# Key factors identified by proteomic analysis in maize (*Zea mays* L.) seedlings’ response to long-term exposure to different phosphate levels

**DOI:** 10.1186/s12953-018-0147-3

**Published:** 2018-11-20

**Authors:** Yanling Sun, Chunhua Mu, Xia Liu

**Affiliations:** 1 Maize Research Institute, Shandong Academy of Agricultural Sciences/National Engineering Laboratory of Wheat and Maize/Key Laboratory of Biology and Genetic Improvement of Maize in Northern Yellow-huai River Plain, Ministry of Agriculture, Jinan, 250100 China; 2 College of Life Sciences, Shandong Normal University, Jinan, 250000 Shandong China

**Keywords:** Proteomics, Maize seedlings, Low-pi tolerance, Pi transporter

## Abstract

**Background:**

Maize seedlings are constantly exposed to inorganic phosphate (Pi)-limited environments. To understand how maize cope with low Pi (LP) and high Pi (HP) conditions, physiological and global proteomic analysis of QXN233 genotype were performed under the long-term Pi starvation and supplementation.

**Methods:**

We investigated the physiological response of QXN233 genotype to LP and HP conditions and detected the changes in ion fluxes by non-invasive micro-test technology and gene expression by quantitative real-time polymerase chain reaction. QXN233 was further assessed using vermiculite assay, and then proteins were isolated and identified by nano-liquid chromatography-mass spectrometry.

**Results:**

A negative relationship was observed between Na^+^ and Pi, and Na^+^ efflux was enhanced under HP condition. Furthermore, a total of 681 and 1374 were identified in the leaves and roots, respectively, which were mostly involved in metabolism, ion transport, and stress response. Importantly, several key Pi transporters were identified for breeding potential. Several ion transporters demonstrated an elaborate interplay between Pi and other ions, together contributing to the growth of QXN233 seedlings.

**Conclusion:**

The results from this study provide insights into the response of maize seedlings to long-term Pi exposure.

**Electronic supplementary material:**

The online version of this article (10.1186/s12953-018-0147-3) contains supplementary material, which is available to authorized users.

## Background

Phosphorus (P) is an essential macronutrient required for normal plant growth and reproduction. P is involved in various metabolism and biological processes, including nucleic acid and protein synthesis, membrane integrity, photosynthesis, respiration, energy metabolism, hormone regulation, stress tolerance, and disease resistance [1, 2]. However, approximately 50% of global agricultural soils suffers from inorganic phosphate (Pi) deficiency [3, 4]. The reason is because plants absorb P in the available form of Pi, which is usually limited in soils due to its immobility and high binding state [5, 6]. Nevertheless, plants use various strategies to adapt to low-Pi (LP) stress, including using free Pi from vacuolar storages [7], deriving organic P from the breakdown of phospholipids, redistributing Pi from old to young tissues, modifying of the root system to increase Pi uptake, obtaining Pi from arbuscular mycorrhizal association [8], and secreting of organic acids and phosphatases to release available Pi from the soil [9–11].

Under Pi deficiency, plants were chlorotic, markedly small and not-well development, presenting necrosis toward the tips of the oldest leaves. Conversely, plants become high and grow up strongly under Pi-sufficient condition [12, 13]. Until now, it has been known that various proteins with Pi transport activity include PHT (phosphate receptor) proteins (involved in Pi uptake from soil and Pi translocation) and the SPX (SYG1, Pho81 and XPR1) domain phosphate transporters [14–18], such as SPX-EXS (ERD1, XPR1, and SYG1) subfamily PHO1 and its homologs (involved in Pi transport from roots to shoots) [19, 20], and the SPX-MFS (major facilitator superfamily) subfamily including VPT1 (vacuolar Pi transporter 1, also named PHT5;1) [21] and OsPHO1;2 (involved in vacuolar Pi transport) [22]. The PHT1 family, which are localized on the plasma membranes, related with highly efficient Pi acquisition from soils, has received considerable attention [6, 10, 23, 24]. Moreover, the PHT2, PHT3, PHT4, and PHT5 family members, which are localized to chloroplasts, mitochondria, Golgi, and vacuole, respectively, and mediated Pi transport across these organelles [25, 26]. In maize (*Zea mays* L.), many *ZmPHTs* genes had been identified, and some transcription factors (TFs) were also found to be involved in the transcriptional regulation of Pi transporters, including myeloblastosis viral oncogene homolog (MYB)-type and basic Helix-Loop-Helix (bHLH)-type [27]. As a member of the MYB superfamily, phosphate starvation response 1 (PHR1) is a major TF involved in Pi signaling, which binds to the PHR1-binding sequence (P1BS) or P1BS-like domain and regulates the expression of many relevant genes under LP condition, including *PHT1*, *Phosphate 1* (*PHO1*), and microRNA399 [27–30]. In *Arabidopsis*, PHR1 interacts with SPX domain proteins in the Pi-dependent manner. Under Pi-sufficient conditions, AtPHR1 has high binding with AtSPX1 to inhibit its Pi starvation-induced targets via P1BS, while weak binding with AtSPX1 to induce the PHR1 target gene expression under Pi-starved conditions [31–34]. In rice, SPXs regulate PHR2 activity in different subcellular levels to regulate Pi signaling. Under high cellular Pi status, as a negative regulator of PHR2, OsSPX4 also function in a Pi dependent way to inhibit PHR2 function; under Pi starvation, OsSPX4 would be degraded, and then OsPHR2 is released into the nucleus and activated Pi-responsive genes expression [35]. Recent report showed that SPX-domain proteins possibly involved in sensing the cellular Pi status by binding to PP-InsPs to regulate Pi homeostasis [36]. However, it needs to be investigated whether SPX-domain proteins are the internal Pi sensors next. Thus, how plants senses the external and internal Pi status remains to be uncover and the sensor have not been verified.

In addition, P nutrition displays various biological interplays with other nutrient pathways, such as the nutrition of nitrogen [37], sulfate [38], zinc [39], iron [40], sucrose, and hormones [41–43], related to the above-mentioned PHT1 members and TFs. Recently, P or Pi was reported to affect arsenic uptake, copper, and aluminum toxicity; citrate synthesis; and starch morphology [44–48]. In maize, global transcriptomic and metabonomic analyses identified multiple Pi-responsive genes that are mostly involved in metabolic pathways and biosynthesis of secondary metabolites and ion transport [49–52]. Nevertheless, the complete Pi signalling and uptake pathways, including how plants sense Pi levels internally by the putative phosphate receptors and the accurate regulatory network of PHT1 transporters, and the cross-talks between Pi and other pathways, are still unclear and need to be further elucidated.

Here, we firstly performed a comparative proteomic analysis of a maize genotype’s response to long-term exposure to external LP and high-Pi (HP) levels. Our goal was to identify several key Pi-responsive proteins related to Pi uptake and transport under different Pi conditions, which may be applied for agricultural production using gene engineering techniques to overexpress one or more of these proteins’ encoding genes in maize. Given the extensive post-transcriptional regulation, the analysis of protein levels may not reflect the transcription levels of genes but closely reflect their functions. Therefore, this study will not only enhance our understanding of the mechanism associated with the response of maize to different Pi levels, but also substantially explore the candidates in breeding the desired gene-modified agricultural crops.

## Methods

### Materials and treatments

QXN233, as a LP-tolerant maize genotype, was reserved in our laboratory [52]. Its seeds were surface sterilized with 10% (*v*/v) NaClO for 5 min, washed with distilled water repeatedly for three times, and then germinated on a wet gauze placed in a plastic square tray for two days. Subsequently, using a quartz sand or vermiculite assay, the uniform seedlings were transferred to a plastic round pot (9 cm × 21 cm × 18 cm, two plants in each pot) filled with quartz sand or vermiculite by irrigating with the normal solution (2 mM Ca(NO_3_)_2_·4H_2_O, 1.25 mM NH_4_NO_3_, 650 μM MgSO_4_·7H_2_O, 650 μM K_2_SO_4_, 500 μM KH_2_PO_4_, 100 μM KCl, 100 μM Fe-EDTA, 10 μM mΜ H_3_BO_4_, 1 μM MnSO_4_, 1 μM ZnSO_4_·7H_2_O, 0.1 μM CuSO_4_·5H_2_O, and 0.5 μM (NH_4_)_6_Mo_7_O_24_). The solutions were applied at 2–3-day intervals. The seedlings were cultivated in a controlled chamber with the temperatures of 28 °C ± 2 °C, relative humidity of 65%, and a photoperiod of 14 h day /10 h night photoperiod. At the three-leaf stage, seedlings were exposed to LP (0 μM KH_2_PO_4_) or HP (3 mM KH_2_PO_4_) treatment, with 500 μM KH_2_PO_4_ as the control. A total of 0.5 L of nutrient solutions containing different Pi levels were applied every two days. After being treated for 20 or 30 days, the plants were photographed and harvested.

For a hydroponic culture, the newly-germinated seedlings were de-embryonated and transferred to moderate grass containers with the normal solution and cultivated in the above chamber. The nutrient solution was changed every day. At the three-leaf stage, seedlings were treated under LP and HP conditions for 12 or 30 h, and then the plants were detected and harvested. A quartz sand or a hydroponic assay was performed due to its less disturbed factors and later easily to rinse the roots.

All the experiments described had at least three biological replicates, and 6–12 plants were used for each replicate.

### Plant growth parameter and chemical analysis

Using a quartz sand assay, at 20 days after the onset of treatment, plant height and leaf length and leaf width were measured by a ruler, and then the length of primary root and number of total roots were measured and counted. Afterward, the fresh weights (FWs) of shoots and roots were each weighed with electronic scales, respectively. Then, the shoots and roots quickly dried at 105 °C for 20 min and then at 85 °C for 48 h or until a fixed weight. Their the dry weights (DWs) of them were recorded and analyzed.

Using a vermiculite assay, the plants were divided into shoots and roots after 30 days of the treatments. On the basis of the plants’ FWs, Pi content was analyzed by using the ammonium molybdate (Mo)-antimony potassium tartrate-ascorbic acid method as described previously [52]. On the basis of the plants’ DWs, Na^+^ and K^+^ contents were analyzed by flame atomic absorption spectroscopy after samples were digested using the HNO_3_–H_2_O_2_ method as previously described [53].

### Malondialdehyde (MDA), compatible solutes contents, and superoxide dismutase (SOD) activity

Using a vermiculite assay, after 20 days of the treatments, the same parts of fresh leaves were harvested from the treated and control samples, quickly frozen in liquid nitrogen and stored at − 80 °C. MDA content was determined by a modified thiobarbituric acid method, proline was examined using the ninhydrin acid reagent method, and the soluble sugar and protein contents were estimated following the protocols described previously [53]. SOD activity was measured spectrophotometrically at 560 nm according to a previously reported method [53].

### Na^+^ and K^+^ fluxes

Using a hydroponic assay, the seedlings were treated for 30 h under LP and HP conditions, and the mature zone of the root was used for the Na^+^ and K^+^ ion flux measurements by non-invasive micro-test technology (NMT) as described previously [53].

### RNA extraction and quantitative real-time polymerase chain reaction (qRT-PCR) analysis

The seedlings were treated for 12 h under LP and HP conditions by using the above-mentioned hydroponic assay. The leaves and roots were collected from the treated and controlled samples, and then they were snap-frozen with liquid nitrogen and ground quickly. RNA was extracted using the E.Z.N.A Plant RNA Kit (OMEGA, USA), and cDNA was synthesized in accordance with the protocol of the 5 × All-in-One RT MasterMix kit (ABM, Canada). qRT-PCR was performed on an ABI 7500 real-time PCR system (ABI, USA) by using the Ultra SYBR Mixture Kit following the manufacturer’s instructions (CWBIO, China). 18 s rRNA was used as an internal reference gene, and the other primers used for qRT-PCR are all shown in Additional file 1: Table S1. The 2^−ΔΔCt^ method was used to calculate for the relative expression of mRNA in the target genes.

### Proteomic data analysis

After the seedlings were treated for 30 days under LP and HP conditions by a vermiculite pot experiment, the leaves and roots were collected and rinsed from the treated and controlled samples, frozen in liquid nitrogen and ground quickly, precipitated in TCA/acetone (1:9), and resuspended in SDT buffer (4% SDS, 100 mM Tris-HCl, 1 mM DTT, pH 7.6). The lysate was sonicated and boiled for 15 min. After centrifugation at 14,000×*g* for 40 min, the supernatant was filtered with 0.22 μm filters, analyzed using the BCA Protein Assay Kit (Bio-Rad, USA), and stored at − 80 °C. A total of 20 μg of proteins for each sample was separated on 12.5% SDS-PAGE, and protein bands were stained with coomassie blue R-250. Then, filter-aided sample preparation digestion was performed as described previously [54]. A total of 100 μg of peptide mixture of each sample was labeled using tandem mass tag (TMT) reagent following the manufacturer’s instructions (Thermo Fisher Scientific). TMT-labeled samples were divided into 10 fractions by using Pierce High pH Reversed-Phase Peptide Fractionation Kit (Thermo scientific).

Each prepared fraction was injected for nano-liquid chromatography–mass spectrometry (LC-MS)/MS analysis. The peptide mixture was loaded onto a reversed phase trap column (Thermo Scientific Acclaim PepMap 100, Nano Viper C18, 100 μm*2 cm) connected with the analytical column of a C18-reversed phase Thermo Scientific Easy Column (length: 10 cm, inner diameter: 75 μm, resin: 3 μm) in buffer solution A (0.1% formic acid) and separated through a linear gradient of buffer solution B (84% acetonitrile and 0.1% formic acid) at a flow rate of 300 nl/min controlled by IntelliFlow technology. The 1.5 h gradient was determined as follows: 0–55% buffer B for 80 min, 55–100% buffer B for 5 min, and then held in 100% buffer B for 5 min. LC-MS/MS analysis was carried out on a Q Exactive mass spectrometer (Thermo Scientific) coupled to Easy nLC (Proxeon Biosystems, now Thermo Fisher Scientific, USA) for 90 min. MS was performed in positive ion mode. The mass spectrometer was acquired with a data-dependent top10 method dynamically choosing the most abundant precursor ions from the survey scan (300–1800 m/z) for higher-energy collisional dissociation (HCD) fragmentation. The automatic gain control target was set to 3e6, the maximum injection time to 10 ms, and the dynamic exclusion duration was 40.0 s. Survey scans were acquired at a resolution of 70,000 at 200 m/z, the HCD spectrum was set to 17,500 at 200 m/z (TMT 6plex), and the isolation width was 2 m/z. The normalized collision energy was 30 eV, and the underfill ratio was 0.1%, which specifies the minimum percentage of the target value likely to be reached at maximum fill time. The instrument was run with peptide recognition mode enabled.

MS/MS spectra were searched using MASCOT engine (version 2.2, Matrix Science, London, UK) embedded into Proteome Discoverer 1.4. Decoy database was used for the false discovery rate (FDR) calculation and data with FDR ≤ 0.01 were filtered. The statistical significance of quantitative data was determined using Student’s *t*-test (*n* = 3, *P* < 0.05), and the different expressed proteins (DEPs) between the LP/HP treatment and control groups were defined as *P* < 0.05 and ratio > 1.2 (up/down regulated). On the basis of Fisher’s exact test, Gene Ontology (GO) enrichment on three ontologies (biological process, molecular function, and cellular component) and Kyoto Encyclopedia of Genes and Genomes (KEGG) pathway enrichment analyses were analyzed to explore the impact of DEPs in cell physiological process.

### Data analysis

Three biological replicates were applied for all the above experiments, and the data were reported as means ± standard error (SEM). The statistical software program used was SPSS version 18.0. All data obtained were subjected to ANOVA, and the significance difference between the two tested groups was determined by *P* value (^*^means *P* < 0.05) on the basis of least-significant difference (LSD) test.

## Results

### Plant growth and the physiological response under LP and HP conditions

**Fig. 1:**
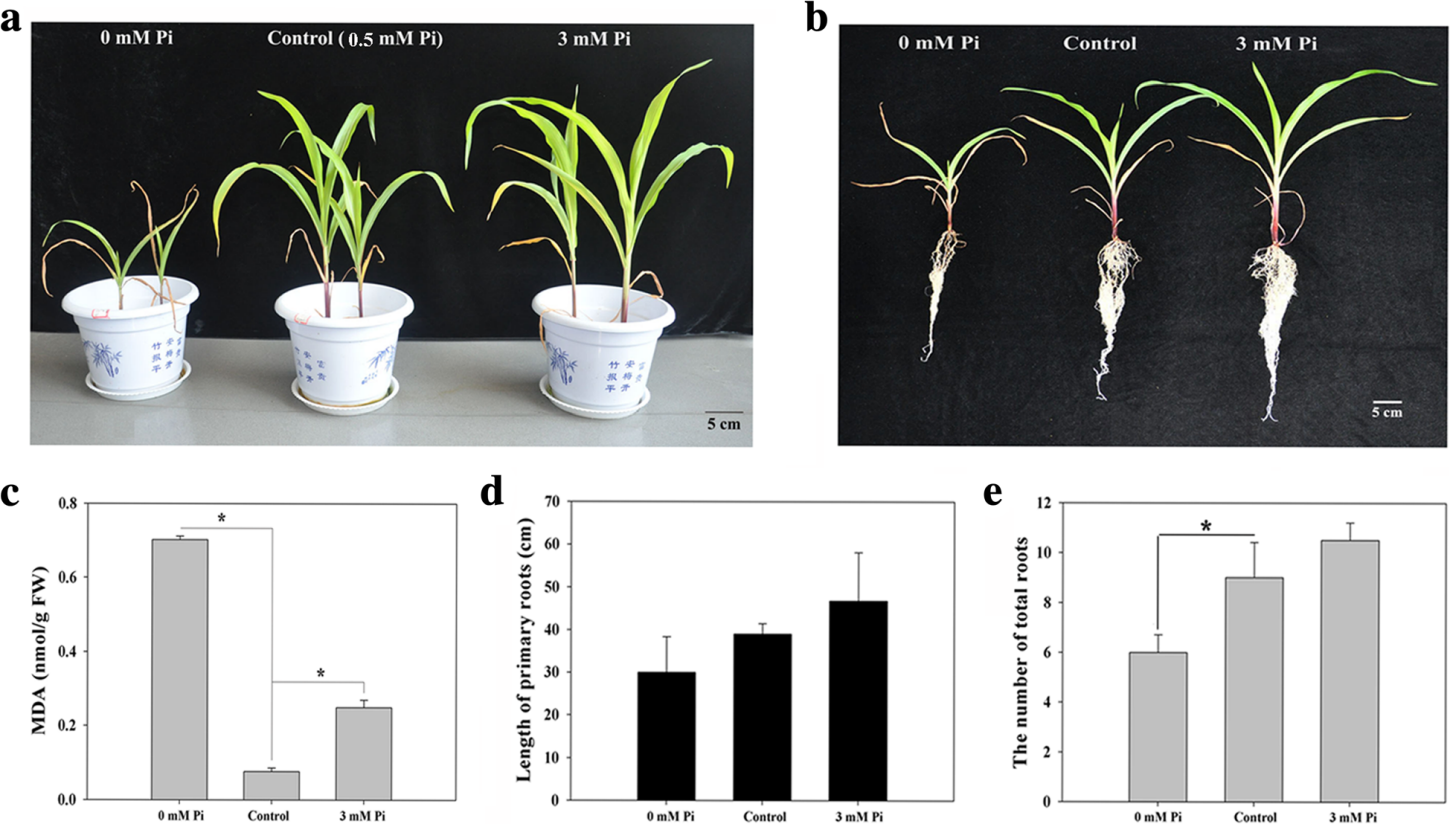
Phenotypic responses and physiological changes when QXN233 was exposed for 20 days to low Pi (LP, 0 mM Pi), control (0.5 mM Pi), and high Pi (HP, 3 mM Pi) conditions using a quartz sand assay. **a** Above-ground phenotypes; **b** under-ground phenotypes; **c** MDA; **d** length of primary roots; **e** the number of total roots. Bar = 5 cm. Values represent means ± SEM of three replicates. Asterisks indicate a significant difference between the two tested groups (LSD test, *P* < 0.05)

**Fig. 2:**
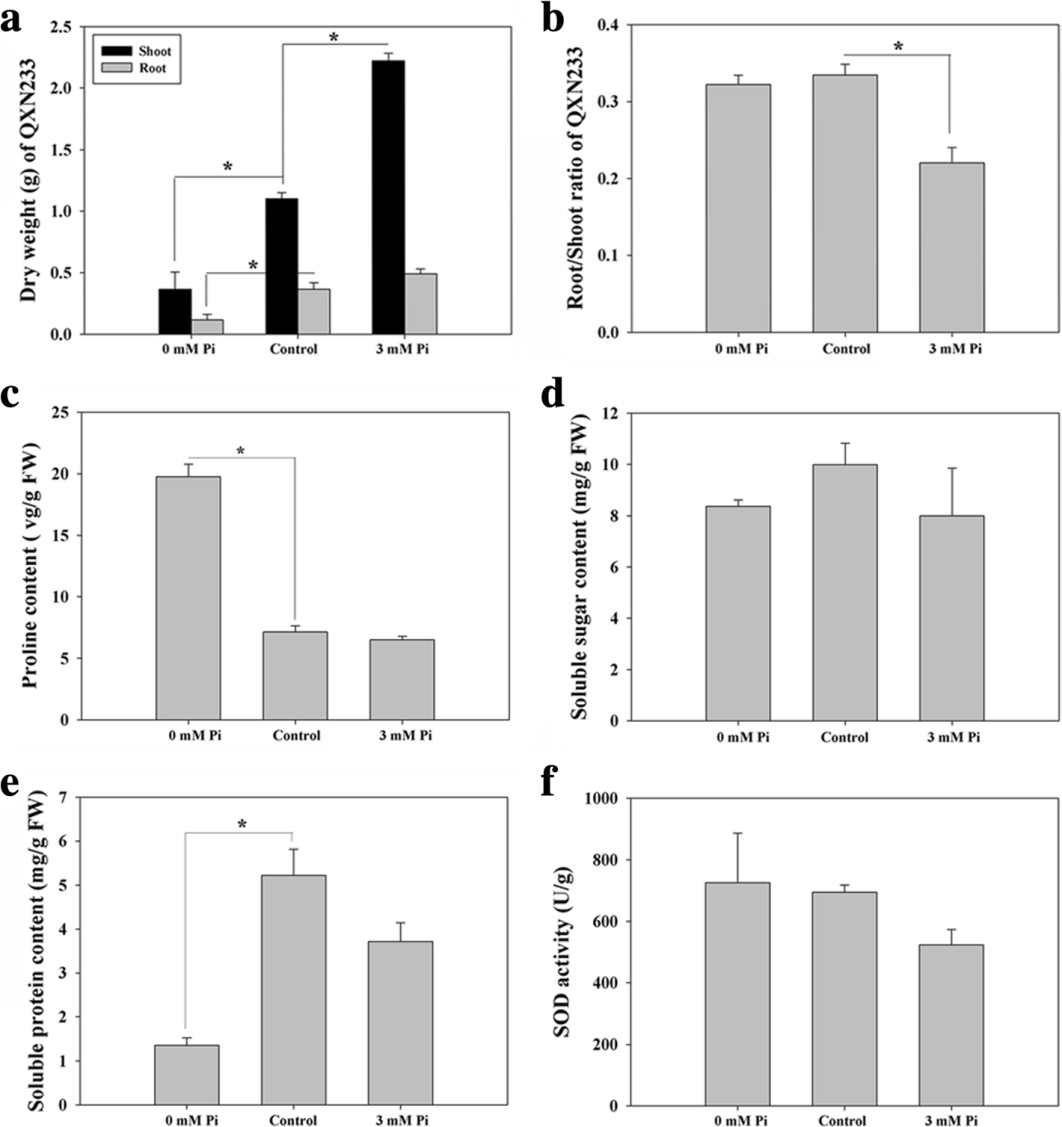
Quantitative analyses of DW, compatible solutes contents and SOD activity in QXN233 after 20 days under LP and HP conditions. **a** DWs of shoots and roots; (**b**) root/shoot ratio; (**c**) proline content; (**d**) soluble sugar content; (**e**) soluble protein content; (**f**) SOD activity. Values represent means ± SEM of three replicates. Asterisks indicate a significant difference between the two tested groups (LSD test, *P* < 0.05)

Under LP condition, QXN233 displayed an obviously inferior phenotype whereas a superior performance under HP condition (Fig. 1a, b; Additional file 1: Figures S1, S2). QXN233 leaves accumulated a large amount of MDA under LP condition, suggesting that severe cell damage occurred in its leaves (Fig. 1c). With the addition of external Pi concentration, the primary root elongated, and the number of total roots also significantly increased (Fig. 1d, e). The plant height, leaf-width and -length, and FWs and DWs increased (Fig. 2a, b; Tables 1, Additional file 1: Table S2). The lowest root/shoot ratio was found in QXN233 under HP condition (Fig. 2b, right), indicating that shoot growth was promoted by sufficient Pi application.

**Table 1:** Quantitative analyses of plant height and the width and length of the longest leaf, and FWs of the shoots and roots in QXN233 after 20 days under 0 mM Pi or 3 mM Pi treatment by a quartz sand assay

QXN233	Plant height (cm)	Width of the longest leaf (cm)	Length of the longest leaf (cm)	FW of shoots (g)	FW of roots (g)
0 mM Pi	17^*^ ± 4.0	2 ± 0.3	23^*^ ± 3.0	2.813^*^ ± 0.1	2.279^*^ ± 0.4
Control	33 ± 6.0	3 ± 0.1	33 ± 4.0	7.688 ± 0.5	5.441 ± 0.3
3 mM Pi	40.5 ± 4.9	4 ± 0.2	46.75^*^ ± 4.6	13.673^*^ ± 1	8.574^*^ ± 0.6

Values represent means ± SEM of three replicates. Asterisks indicate a significant difference between the Pi-treated and control group (*t*-test, *P* < 0.05)

In addition, when exposed to LP condition, QXN233 accumulated higher proline content but less soluble protein content than those of the control or HP group, and no significant difference was found between soluble sugar content and SOD activity among the three Pi levels (Fig. 2c-f).

### Na^+^, K^+^, and pi distributions and relevant gene expression under LP and HP conditions

**Fig. 3:**
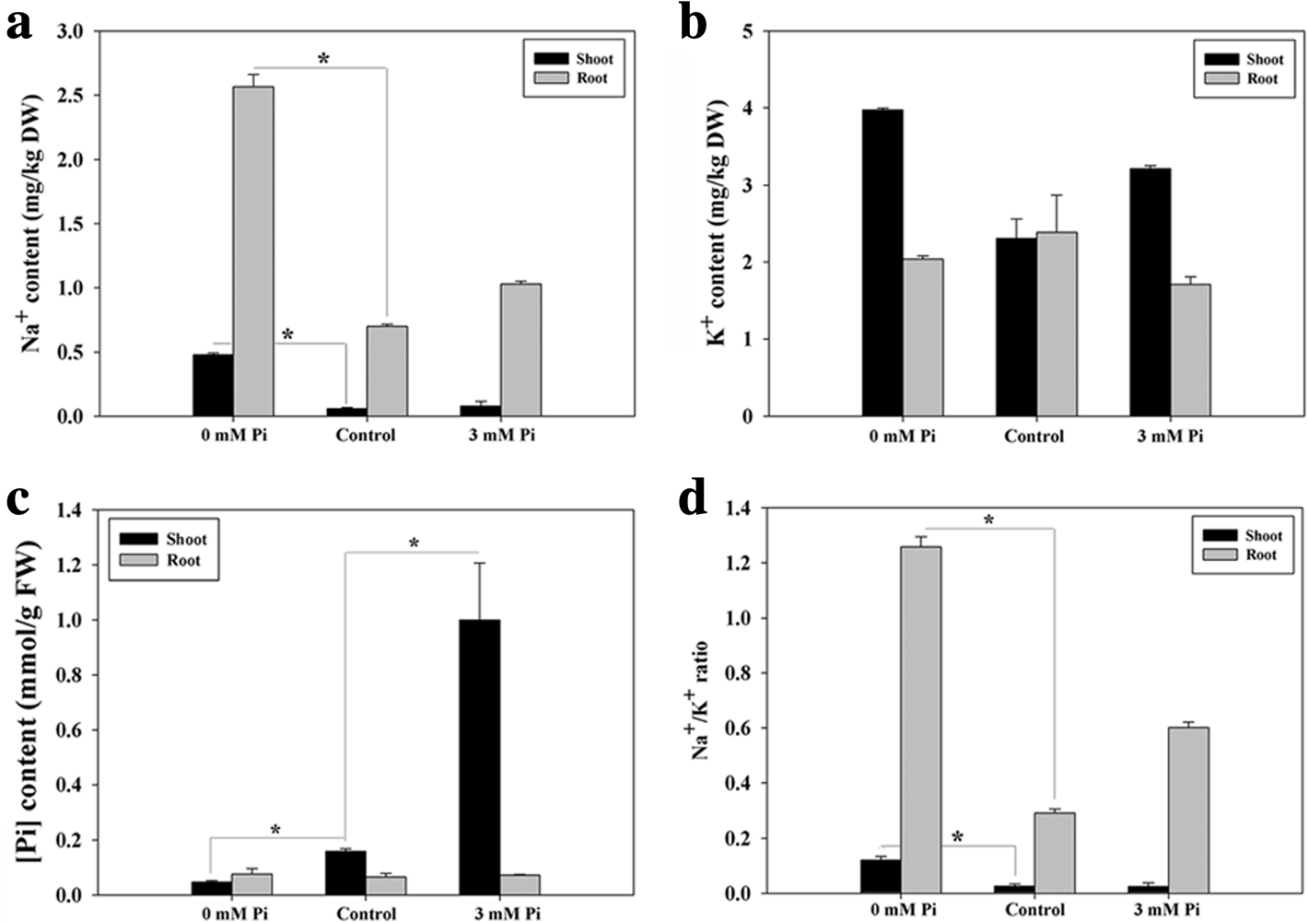
Changes in Na^+^ (**a**), K^+^ (**b**), and Pi (**c**) ion content and Na^+^/K^+^ ratio (**d**) in QXN233 exposed for 30 days to the control, LP and HP conditions. Values represent means ± SEM of three replicates. Asterisks indicate a significant difference between the two tested groups (LSD test, *P* < 0.05)

To further investigate the ion distributions of QXN233 seedlings under different Pi conditions, Na^+^, K^+^, and Pi contents were measured in shoots and roots. Under LP condition, the Na^+^ content in the shoots and especially, the roots increased, whereas no significant difference was found in terms of K^+^ content, resulting in a high Na^+^/K^+^ ratio (Fig. 3a, b, d). In contrast to LP stress, a low Na^+^ content and Na^+^/K^+^ ratio showed in plants under HP condition, especially in shoots, although no significant change compared to the control (Fig. 3a, d). Obviously, the Pi content in QXN233 shoots increased as the external Pi supplement increased, and the highest content was observed at the 3 mM Pi condition (Fig. 3c), indicating a strong Pi uptake and assimilation by shoot cells. Of note, low Na^+^ was found in shoots under HP condition (Fig. 3a, c), indicating a negative relationship between Na^+^ and Pi in the shoots.

**Fig. 4:**
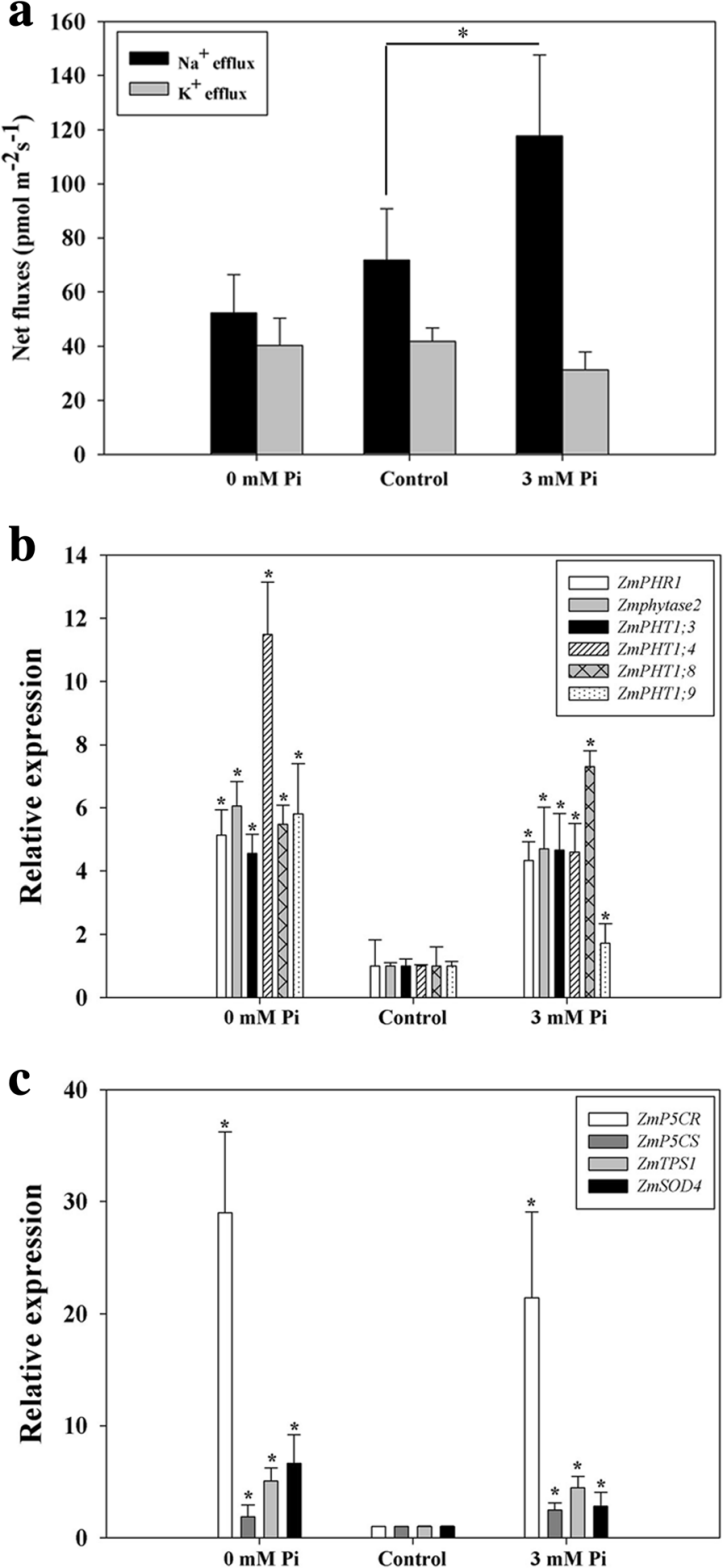
Changes in net Na^+^ and K^+^ fluxes (**a**) and gene expression analysis (**b** and **c**) of QXN233 under LP and HP conditions. Ion fluxes were measured in the root mature zones of QXN233 exposed to control, LP and HP conditions for 30 h, and the relative gene expressions were detected by qRT-PCR in QXN233 roots after 12 h under LP and HP treatments. Values represent means ± SEM of three replicates. Asterisks indicate a significant difference between the two tested groups (LSD test, *P* < 0.05)

Moreover, Na^+^ and K^+^ ion fluxes were detected using NMT at 30 h after QXN233 roots were exposed to LP and HP conditions, respectively. The result showed that a high Na^+^ efflux emerged in the roots under HP condition (Fig. 4a), implying that high Na^+^ exclusion was induced by HP supplementation. Furthermore, the Pi-responsive genes, including the early gene *ZmPHR1* and the Pi transporters *ZmPHT1;3* (GRMZM2G112377), *ZmPHT1;4* (GRMZM2G170208), *ZmPHT1;8* (GRMZM2G045473), *ZmPHT1;9* (GRMZM2G154090), and *Zmphytase2* were all upregulated under LP and HP conditions (Fig. 4b), especially for *ZmPHT1;9* (GRMZM2G154090), as the encoding gene of Q49B46 detected in below proteomic analysis. This result suggested that these genes play important roles in Pi uptake and homeostasis. The gene encoding proline synthesis *Zea mays pyrroline-5-carboxylate reductase* (*ZmP5CR)* was also upregulated under both LP and HP conditions, which is similar to the other related genes, including *Zea mays pyrroline-5-carboxylate synthetase* (*ZmP5CS)*, as well as *Zea mays trehalose-6-phosphate synthase* (*ZmTPS1*) and *Zea mays superoxide dismutase* (*ZmSOD4)*, which encode enzymes involved in the trehalose and SOD biosynthesis pathway, respectively (Fig. 4c), This finding implied that these genes play an important role for osmotic adjustment under Pi starvation and excess Pi supplementation providing nutrition also induces slight stress to plant cells.

### Proteome analysis of QXN233 under LP and HP conditions

**Fig. 5:**
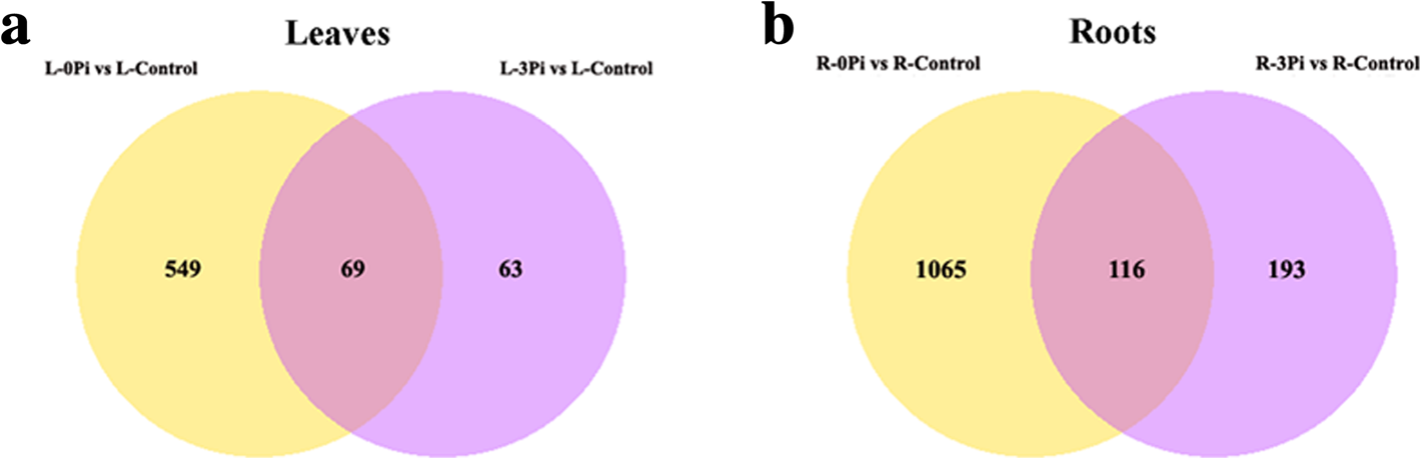
Distribution of DEPs in leaves (**a**) and roots (**b**) under LP and HP conditions

**Fig. 6:**
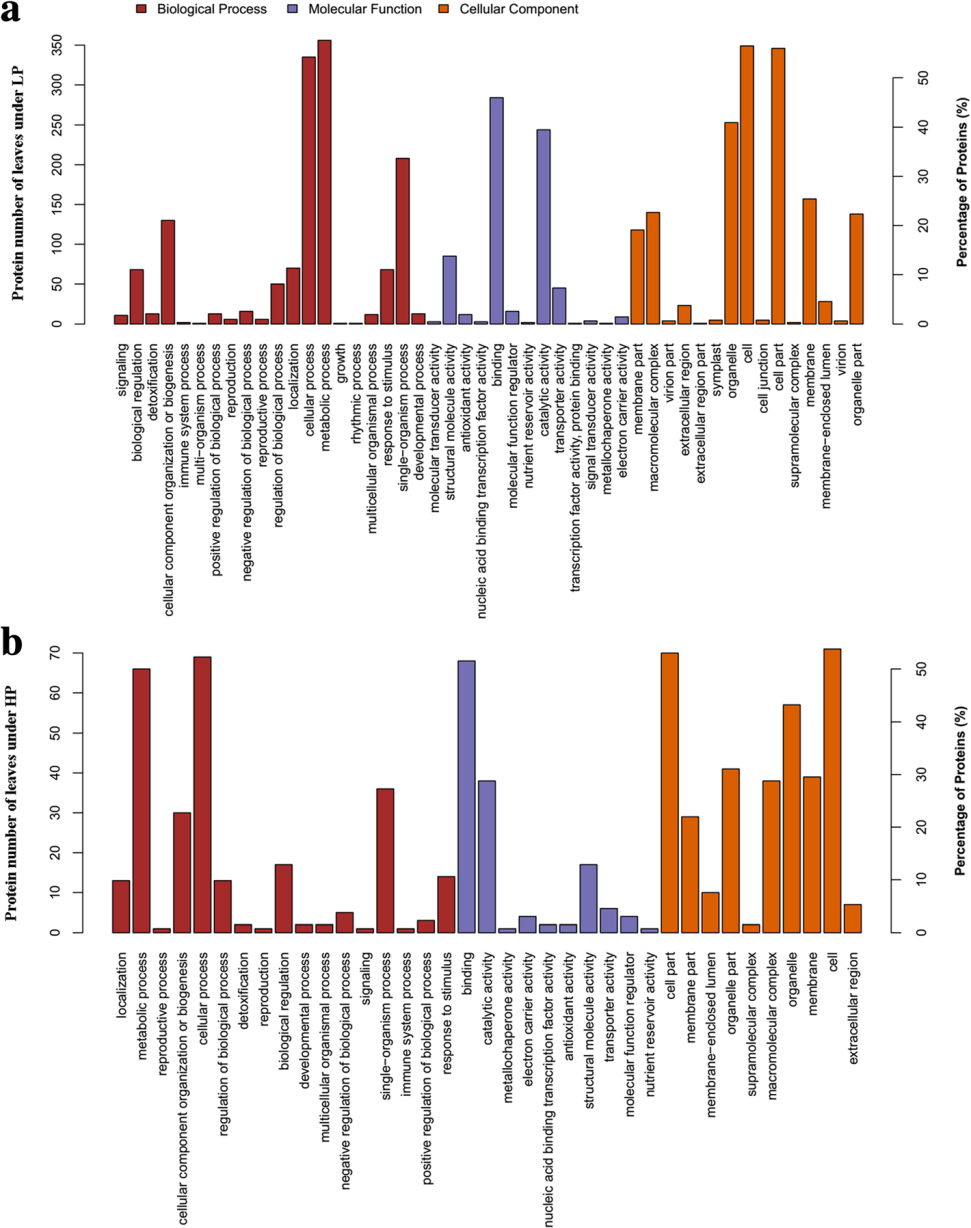
GO categories of DEPs on the basis of GO enrichment analysis in leaves under LP (**a**) and HP (**b**) conditions

**Fig. 7:**
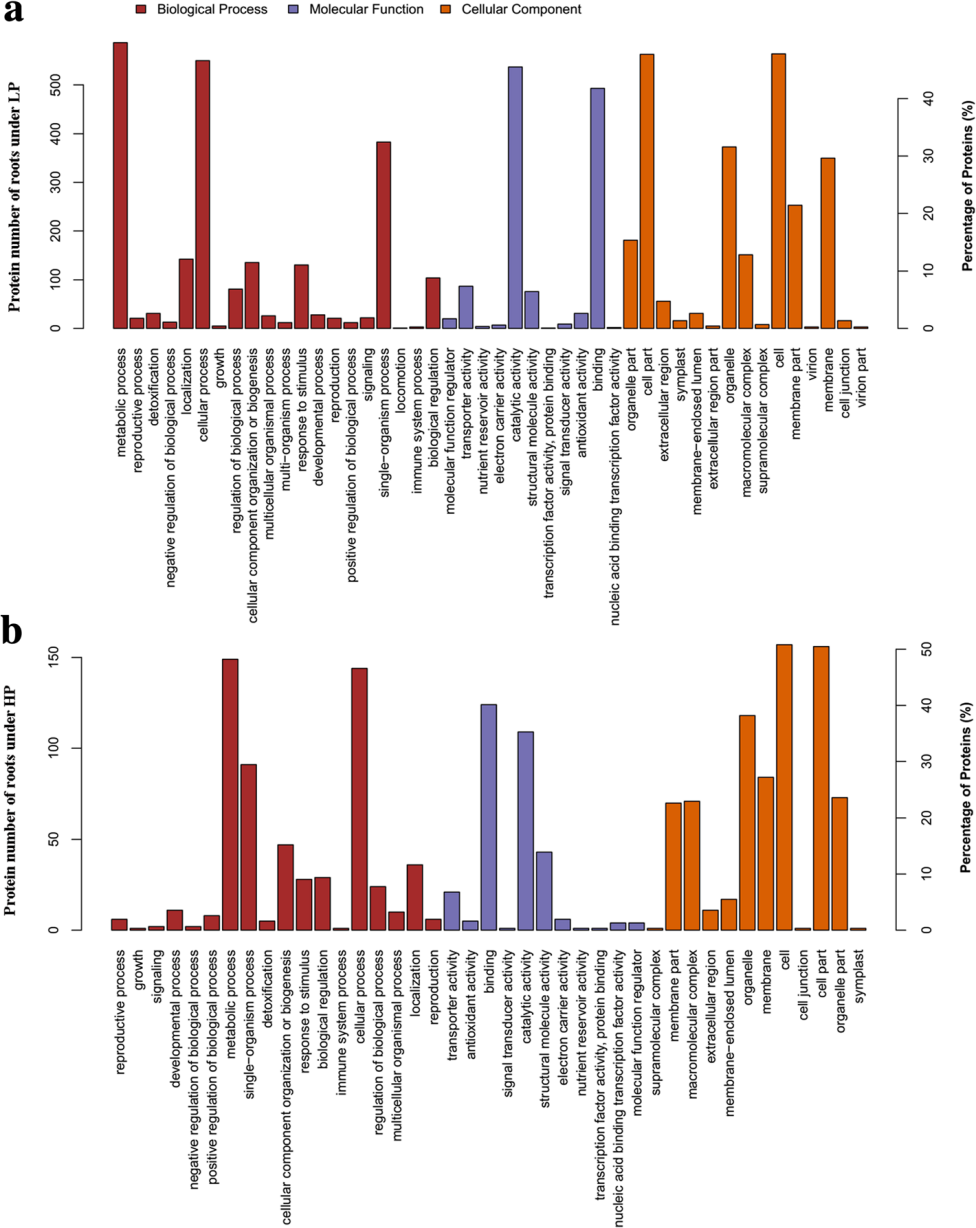
GO categories of DEPs on the basis of GO enrichment analysis in roots under LP (**a**) and HP (**b**) conditions

**Fig. 8:**
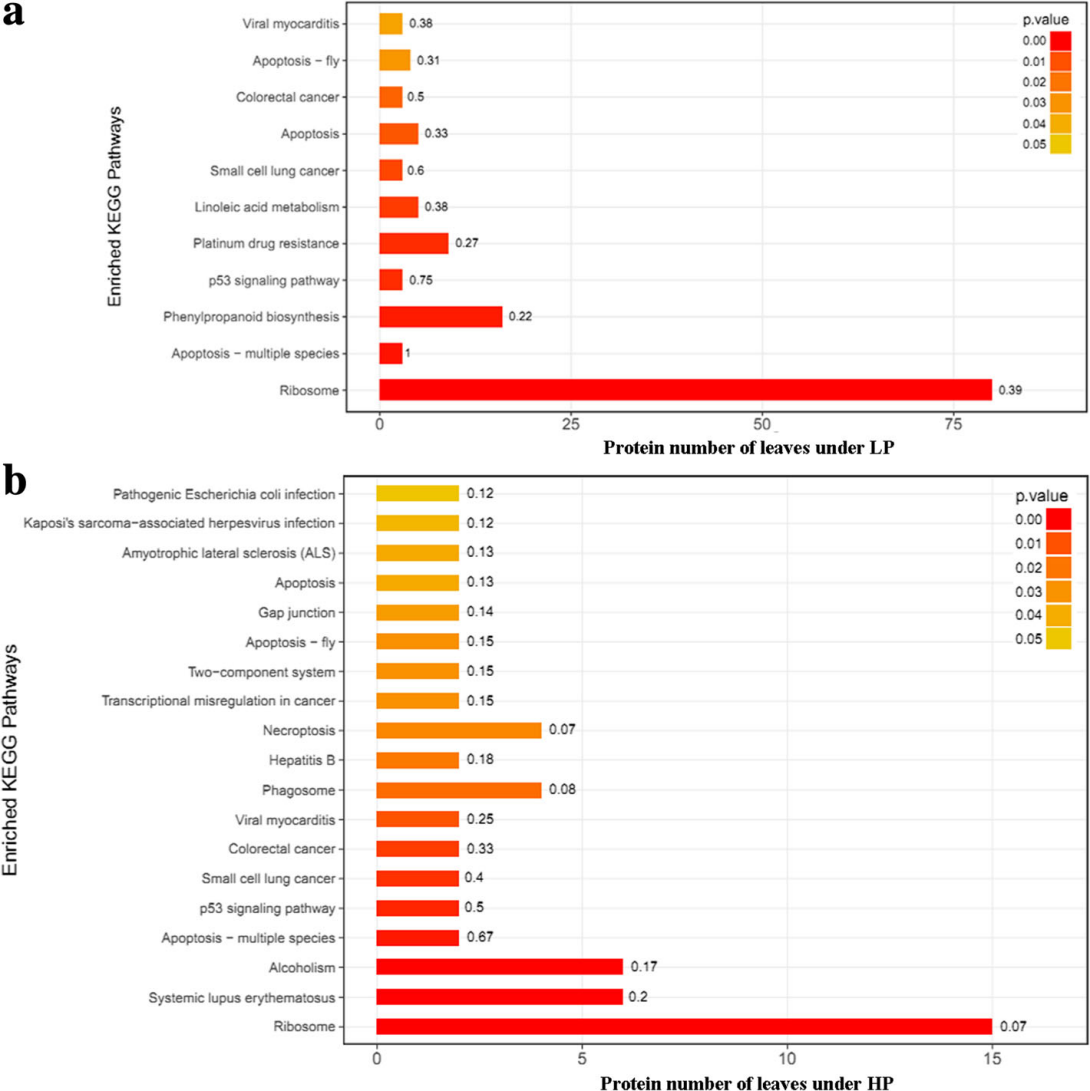
KEGG pathways of DEPs on the basis of KEGG enrichment analysis in leaves under LP (**a**) and HP (**b**) conditions

**Fig. 9:**
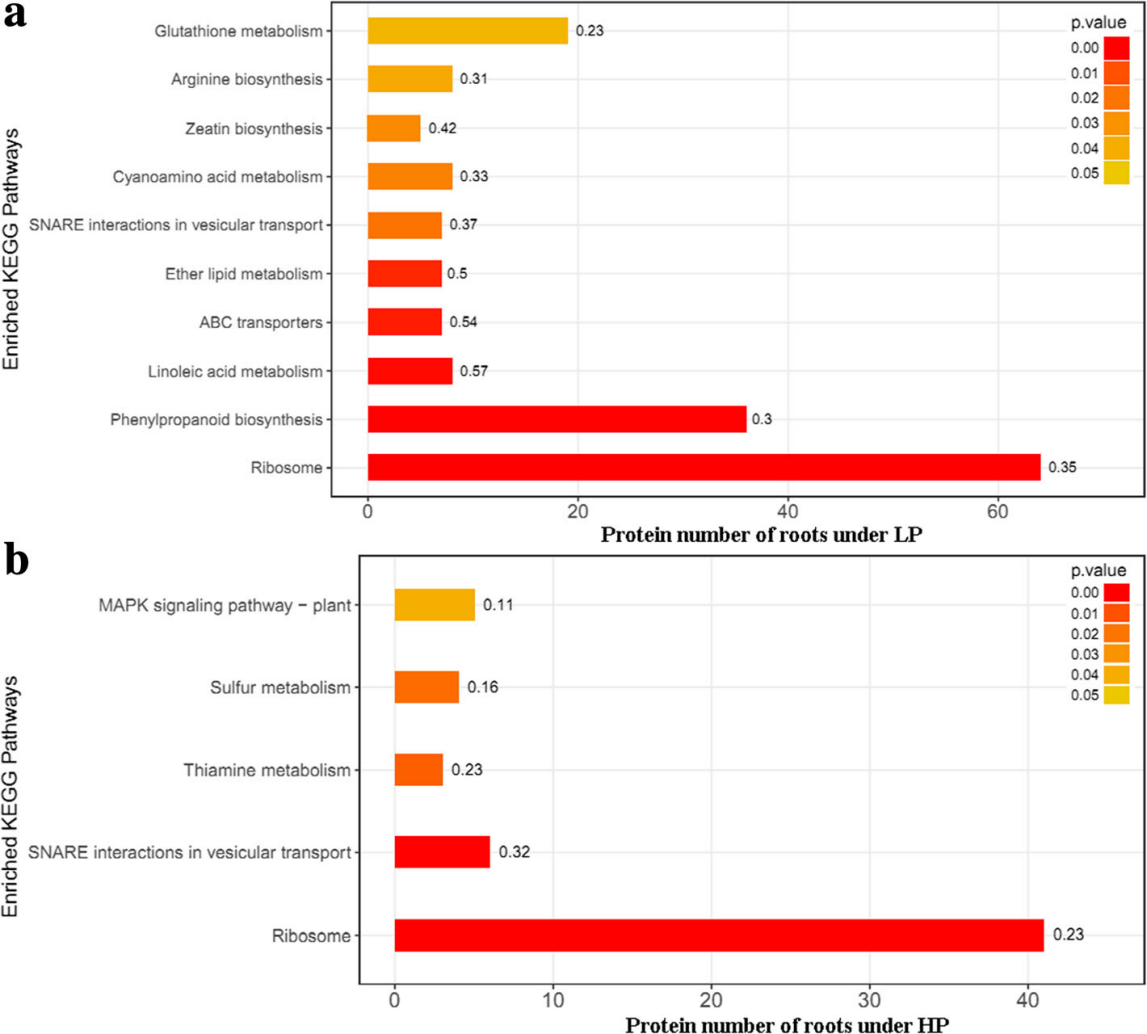
KEGG pathways of DEPs on the basis of KEGG enrichment analysis in roots under LP (**a**) and HP (**b**) conditions

**Fig. 10:**
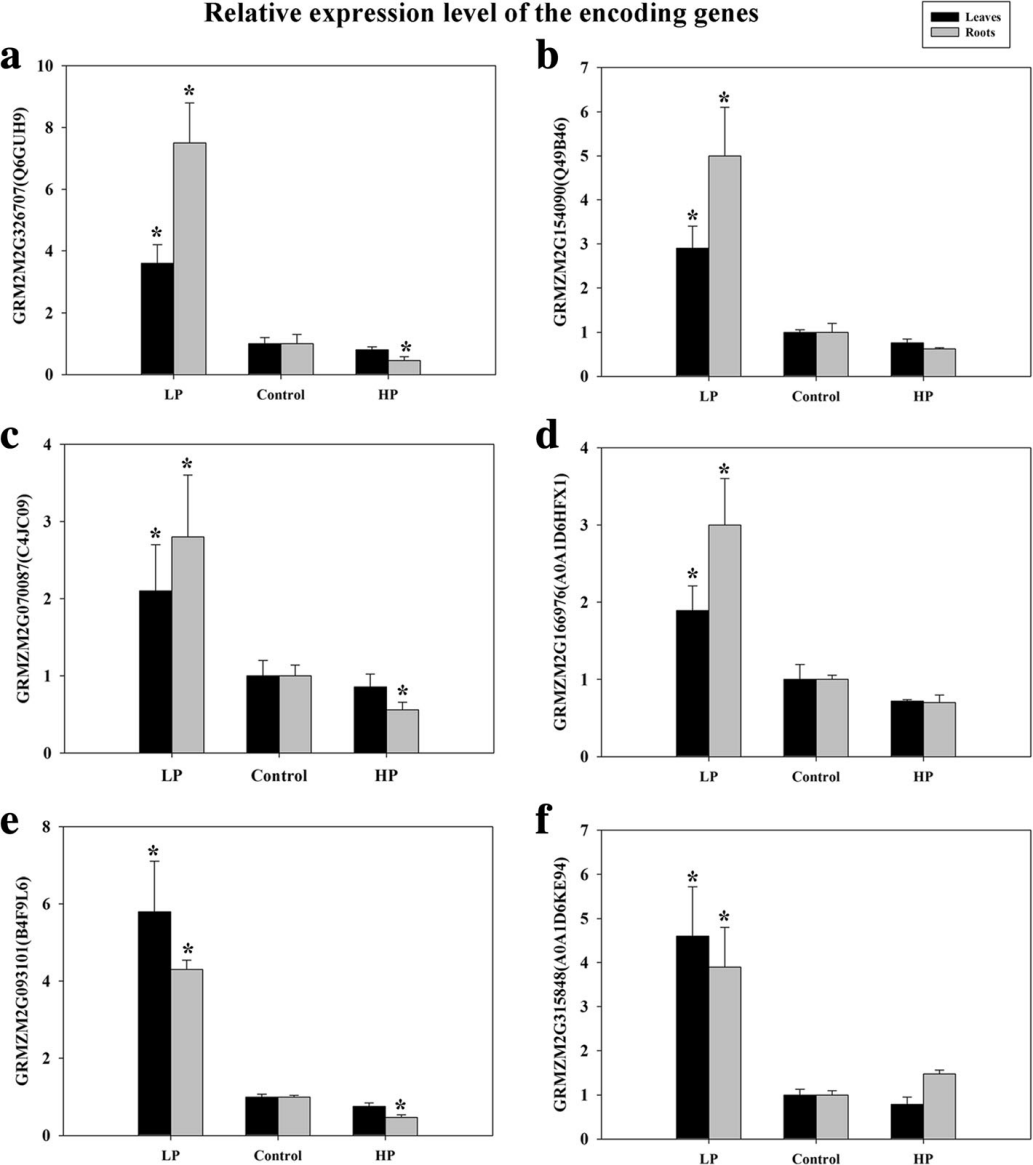
Validation of the gene expression profiles from the DEPs identified by proteomic analysis using qRT-PCR. Relative expressions of six tested Pi-responsive genes in leaves and roots are shown under LP and HP conditions. **a** GRM2M2G326707 (Q6GUH9); (**b)** GRMZM2G154090 (Q49B46); (**c**) GRMZM2G070087 (C4JC09); (**d)** GRMZM2G166976 (A0A1D6HFX1); (**e**) GRMZM2G093101 (B4F9L6); (**f)** GRMZM2G315848 (A0A1D6KE94). Values represent means ± SEM of three replicates. Asterisks indicate a significant difference between the two tested groups (LSD test, *P* < 0.05)

To gain more insights into the regulated mechanism of QXN233 response to long-term LP or HP conditions, we performed quantitative proteomic analysis in its leaves and roots by TMT technology. Compared with normal condition, the DEP profiles under LP or HP condition were identified. A total 681 DEPs in the leaves and 1374 DEPs in the roots, and 69 overlapped DEPs in the leaves and 116 overlapped DEPs in the roots were generated (Fig. 5; Additional file 1: Tables S3, S4). Furthermore, under LP and HP conditions, GO analysis in QXN233 both showed that the main biological processes were involved in the metabolic and cellular process, and the main molecular function related to the binding and the main catalytic activity (Figs. 6, 7). KEGG analysis also revealed that the major pathways were both associated with ribosomes in the leaves and roots of QXN233 under LP or HP condition, indicating that Pi plays a vital role in the ribosomes (Figs. 8, 9). Moreover, some important encoding genes of DEPs were confirmed by qRT-PCR, most of them were upregulated under LP condition while downregulated or no significant change under HP condition (Fig. 10). Thus, the level of their transcript in leaves or roots corresponded to the proteomic analysis of maize in response to LP and HP conditions (Fig. 10; Table 2), implying that the proteomic analysis is reliable.

**Table 2:** Differentially expressed transporters of QXN233 both identified under LP and HP compared with the normal condition by the proteomic analysis (Ratio |0 Pi or 3 Pi/Control| > 1.2 and *P* value < 0.05)

Accession number	Protein description	L-(0Pi/Control)	L-(3Pi/Control)	R-(0Pi/Control)	R-(3Pi/Control)
Pi-related transporters					
Q6GUH9	Phosphate transport protein	1.53	–	1.32	0.79
Q49B46	Inorganic phosphate transporter 1	1.49	–	1.94	0.79
C4JC09	Phosphate transporter protein 9	–	–	1.25	0.62
B4F9L6	Purple acid phosphatase	2.2	–	1.84	0.62
A0A1D6KE94	Purple acid phosphatase	2.03	–	1.67	0.62
Carbohydrate transporters					
C0PHL2	Monosaccharide transporter 1	1.72	–	1.33	0.83
K7TK04	Carbohydrate transporter/ sugar porter	–	–	4.655	0.61
A0A1D6EP34	Sugar transport protein 14	–	–	1.4	0.78
B6TZY0	Facilitated glucose transporter member 8	–	–	1.38	0.77
ABC transporters					
A0A1D6NEN5	ABC transporter B family member 9	1.29	–	1.72	0.79
B4FBM3	ABC transporter G family member 6	–	–	1.31	0.82
Other transporters					
C0P6N0	Calcium load-activated calcium channel	0.74	–	0.73	1.65
Q6I681	Ascorbate-specific transmembrane electron transporter 1	–	–	1.39	0.83

### DEPs involved in various metabolic and signal pathways

On the basis of the predicted functions, many DEPs were presented and classified into different groups (Additional file 1: Table S3), including metabolism, photosynthesis, ATP metabolism, transcriptional regulators, translation, cell growth, phytohormone regulation, oxidation-reduction process, and stress response. Compared with HP condition, we found that the majority of DEPs involved in metabolism, photosynthesis, and ATP metabolism were highly expressed in QXN233 leaves under LP stress, suggesting that Pi deficiency has important effects on these aspects. Similarly, under LP stress, many DEPs associated with oxidation-reduction and stress response were both upregulated in the leaves and roots, including SOD, peroxidase, heat shock 70 kDa protein, and aquaporin, suggesting possible strategies in response to Pi deficiency. Moreover, some DEPs involved in the phytohormone regulation, were increased in leaves or roots, including abscisic acid, brassinosteroid, and gibberellic acid, were increased in the leaves or roots, suggesting that phytohormones participate in the LP response. By contrast, DEPs associated with 40S and 60S ribosomal assembly were downregulated in the leaves and roots under LP stress (Additional file 1: Tables S3, S4), indicating a decrease in ribosomal activity due to Pi deficiency. In addition, the expression patterns of DEPs related to cell growth, including K7W5K6 (pectinesterase), A0A077D360 (cellulose synthase) and B6TH29 (cell division protein ftsZ) which were downregulated in the leaves under LP stress (Additional file 1: Table S3), probably led to the weak growth performance under Pi deficiency, were intricate and need to be studied further.

### Key DEPs related with pi transport under LP and HP conditions

Under LP condition, Q6GUH9 (phosphate transport protein), Q49B46 (inorganic phosphate transporter 1), and C4JC09 (phosphate transporter protein 9) were upregulated in the leaves or roots but downregulated under HP condition (Table 2). Moreover, two purple acid phosphatases (PAPs), B4F9L6 and A0A1D6KE94, presented the same trends as the above Pi transporters, which freed Pi from phytase-P (Table 2), suggesting that these proteins play vital roles in Pi assimilation, transport and remobilization. Except for the common DEPs, B6SYB8 (Pi starvation-induced protein, fold change = 1.29 in leaves), A0A1D6QB24 (phosphate transporter 2, fold change = 2.4 in roots), and A0A1D6HFX1 (SPX domain-containing membrane protein, fold change = 1.35 in roots) were also uniquely increased under LP condition (Table 3), suggesting their contribution to Pi acquisition. Moreover, some PAPs, A0A1D6EDB6 (fold change =1.57 in leaves) and K7TEL4 (fold change = 2.14 in roots), and B6TWW2 (fold change = 0.63 in roots) were also up−/downregulated accordingly in LP/HP condition, but B4FR72 (fold change = 0.75 in roots) requires further study (Table 3).

**Table 3:** Differentially expressed transporters of QXN233 identified under LP or HP condition compared with the normal condition by the proteomic analysis (Ratio |0 Pi or 3 Pi/Control| > 1.2 and *P* < 0.05)

Accession number	Protein description	L-(0Pi/Control)	L-(3Pi/Control)	R-(0Pi/Control)	R-(3Pi/Control)
Pi-related transporters					
B6SYB8	Pi starvation-induced protein	1.29	–	–	–
A0A1D6QB24	Phosphate transporter 2	–	–	2.4	–
A0A1D6HFX1	SPX domain-containing membrane protein	–	–	1.35	–
A0A1D6EDB6	Purple acid phosphatase	1.57	–	–	–
K7TEL4	Purple acid phosphatase	–	–	2.14	–
B4FR72	Purple acid phosphatase	–	–	0.75	–
B6TWW2	Purple acid phosphatase	–	–	–	0.63
Carbohydrate transporters					
A0A1D6MQM6	Carbohydrate transporter/sugar porter/transporter	1.8	–	–	–
B6TCP1	Carbohydrate transporter/sugar porter/transporter	1.46	–	–	–
A0A1D6MV11	Sucrose transporter 4	–	–	1.31	–
A0A1D6IFG4	Sugar carrier protein C	1.35	–	–	–
B6TEX4	Sugar transport protein 5	0.78	–	–	–
A0A1D6MLZ7	Plastidic glucose transporter 4	–	–	1.25	–
A0A1D6IWW8	Putative polyol transporter 1	1.51	–	–	–
A0A1D6IHY2	Putative polyol transporter 1	–	–	1.45	–
A0A1D6IHX5	Putative polyol transporter 1	–	–	1.38	–
A0A1D6GE23	D-Xylose-proton symporter-like 3 chloroplastic	–	–	1.37	–
Cation transporters					
A0A096PXB4	Vacuolar cation/proton exchanger 3	2.61	–	–	–
B4FUC4	Plasma membrane-associated cation-binding protein 1	1.56	–	1.77	1.52
A0A1D6HHV5	Cation/H(+) antiporter 1	1.45	–	–	–
C0PHC1	Chloride channel protein	1.42	–	1.28	–
A0A1D6FLY1	Calcium permeable stress-gated cation channel 1	–	–	1.21	–
A0A1D6N218	Potassium transporter 3	–	–	1.42	–
W5UB74	Potassium transporter	–	–	1.26	–
A0A1D6DSW6	K(+) efflux antiporter 2 chloroplastic	1.68	–	–	–
A0A1D6JIE4	K(+) efflux antiporter 2 chloroplastic	1.32	–	–	–
B6T7A1	Voltage-gated potassium channel beta subunit	–	–	0.8	–
A0A1D6KAA6	Protein NRT1/PTR family 8.3	–	–	1.35	–
A0A1D6N629	Protein NRT1/PTR family 5.10	–	–	0.68	–
ABC transporters					
A0A1D6NEQ4	ABC transporter B family member 9	–	–	2.34	–
A0A1D6G2R7	ABC transporter B family member 9	–	–	1.55	–
A0A1D6MRC7	ABC transporter B family member 21	1.38	–	–	–
A0A1D6J0E8	ABC transporter B family member 19	–	–	1.83	–
A0A1D6J8P3	ABC transporter B family member 15	–	–	1.67	–
A0A1D6FYX8	ABC transporter G family member 40	–	–	1.79	–
A0A1D6Q0P9	ABC transporter A family member 7	–	–	1.68	–
A0A1D6Q0P8	ABC transporter A family member 7		–	1.3	–
A0A1D6E1K8	ABC transporter C family member 9	–	–	1.51	–
A0A1D6FF20	ABC transporter C family member 3	–	–	1.49	–
A0A1D6EEY6	ABC transporter C family member 4	–	–	1.47	–
K7VCA6	ABC transporter C family member 4	–	–	1.43	–
A0A1D6J4B3	ABC transporter C family member 4	–	–	1.37	–
Amino acid transporters					
A0A1D6KGL1	Cationic amino acid transporter 4 vacuolar	–	–	1.45	–
A0A1D6KGL3	Cationic amino acid transporter 4 vacuolar	1.36	–	–	–
A0A097ETZ6	Urea transporter	–	–	0.82	–
B4FYF5	Lysine histidine transporter 2	–	–	0.76	–
Vesicle transporters					
A0A1D6G304	Vesicle transport v-SNARE 12	–	–	1.44	1.27
B4FFY7	Endoplasmic reticulum vesicle transporter protein	–	–	0.81	–
A0A1D6E9B4	Endoplasmic reticulum vesicle transporter protein	1.28	–	–	–
Metal transporters					
A0A1D6PHW0	Metal transporter Nramp3	–	–	1.35	–
A0A1D6M2W3	Heavy metal transport/detoxification superfamily	0.79	–	–	–
Other transporters					
K7UFL6	Putative glycerol-3-phosphate transporter 1	–	–	2.15	–
A0A1D6JAP3	Putative sphingolipid transporter spinster homolog 2	–	–	1.25	–
A0A1D6KXB5	Organic cation/carnitine transporter 7	1.45	–	1.92	–
A0A096TR23	Molybdate transporter 2	2.08	–	1.54	–
E3UJZ2	Putative metal-nicotianamine transporter YSL7	1.27	–	–	–
A0A1D6PKM7	Boron transporter 4	0.77	–	–	–

Furthermore, searching from Uniprot (https://www.uniprot.org/) and NCBI (https://www.ncbi.nlm.nih.gov/) database, some DEPs’encoding genes were analyzed, including Q6GUH9 (*ZmPHT1*, GRMZM2G326707), Q49B46 (*ZmPHT1;9*/*ZmPHT2*, GRMZM2G154090), C4JC09 (*ZmPHT9*, GRMZM2G070087), B4F9L6 (*ZmPAP10*, GRMZM2G093101), A0A1D6KE94 [nucleotide pyrophosphatase/phosphodiesterase (*ZmNPP*), GRMZM2G315848], and A0A1D6HFX1 (*ZmSPX*, GRMZM2G166976). The expression levels of these genes were examined by qRT-PCR. The results showed that *ZmPHT1, ZmPHT2*, and *ZmPHT9* increased in leaves or roots under LP condition while decreased under HP condition, consistent with the results of proteomic analysis (Fig. 10a-c; Table 2). Similarly, the expression patterns of *ZmPAP10, ZmNPP*, and *ZmSPX* were also nearly in accordance with the changing trend of the relevant protein levels by proteomic analysis (Fig. 10d-f; Table 2).

### Other transporters identified under LP and HP conditions

Aside from the above transporters, many other transporters, including carbohydrate transporters, cation transporters, and ATP-binding cassette (ABC) transporters, were also identified. Similarly, some of them presented opposite expression patterns under LP and HP conditions (Table 2). For carbohydrate transporters, C0PHL2 (monosaccharide transporter1), K7TK04 (carbohydrate transporter/sugar porter), A0A1D6EP34 (sugar transport protein 14) and B6TZY0 (facilitated glucose transporter member 8), were increased under LP condition but suppressed under HP condition in the leaves or roots, indicating that these transporters regulated sugar metabolism when maize seedlings adapted to different Pi levels (Table 2). A0A1D6MQM6 and B6TCP1 (carbohydrate transporter/sugar porter/transporter), and A0A1D6IFG4 (sugar carrier protein C), A0A1D6MV11 (sucrose transporter 4), A0A1D6MLZ7 (plastidic glucose transporter 4), and three putative polyol transporters, were also induced under LP condition (Table 3).

For the cation transporters, two K^+^ transporters, A0A1D6N218 (fold change = 1.42 in roots) and W5UB74 (fold change = 1.26 in roots), were elevated under LP condition, but B6T7A1 (voltage-gated K^+^ channel beta subunit) and A0A1D6DSW6 and A0A1D6JIE4 (two K^+^ efflux antiporters, chloroplastic), responsible for K^+^ transport and exclusion, were also induced. Moreover, two nitrate transporters, A0A1D6KAA6 (protein NRT1/PTR family 8.3, fold change = 1.35 in roots) and A0A1D6N629 (protein NRT1/PTR family 5.10, fold change = 0.68 in roots), were detected in roots under LP condition. Many ABC transporters were also significantly altered in roots. Among them, A0A1D6NEN5 (ABC transporter B family member 9) and B4FBM3 (ABC transporter G family member 6) showed opposite expression patterns in roots’ response to LP and HP conditions (Tables 2, 3).

Interestingly, several other transporters were also identified. C0P6N0 (calcium load-activated calcium channel) was both downregulated in the leaves and roots under LP condition but upregulated in the roots under HP condition. Q6I681 [ascorbate (As)-specific transmembrane electron transporter 1] was up- and downregulated in roots under LP and HP conditions, respectively. Under LP condition, A0A096TR23 [molybdate (Mo) transporter 2] was highly expressed in the leaves and roots and A0A1D6PKM7 [boron (B) transporter 4] was lowly expressed in the leaves. These results indicated that high As and Mo and low B contents are required by plants under Pi deficiency (Table 3).

## Discussion

**Fig. 11:**
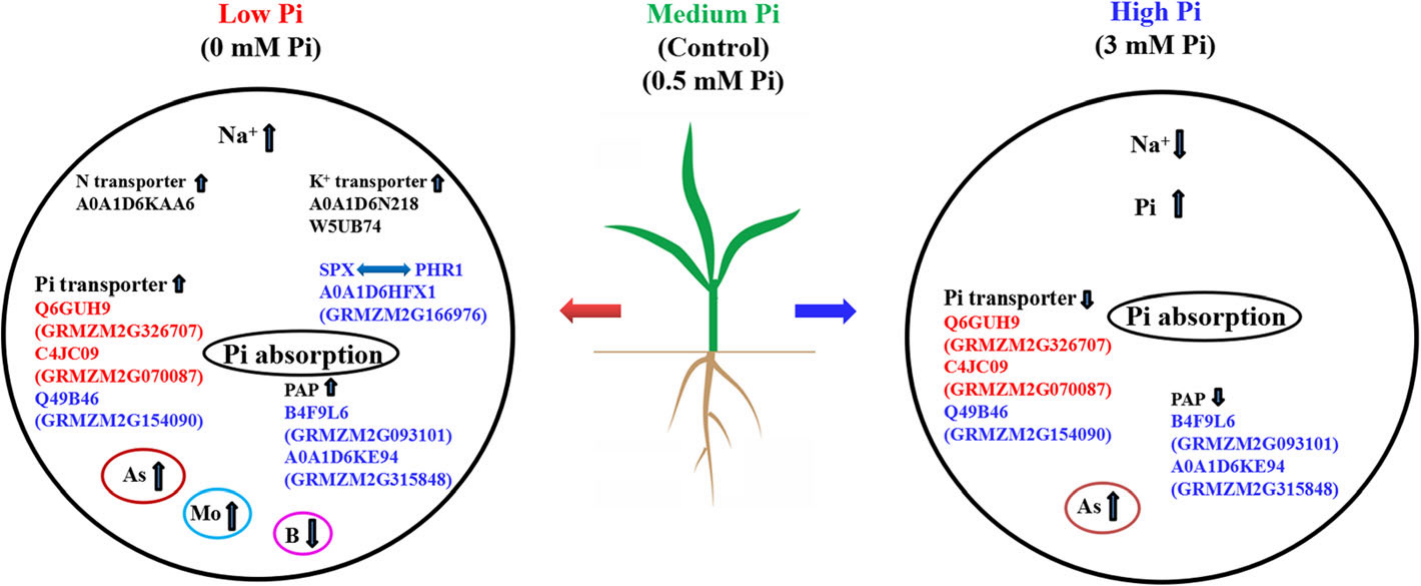
Schematic representation of the regulated mechanism of QXN233 in response to long-term exposure to LP and HP conditions. Pi transporter proteins Q6GUH9, Q49B46, and C4JC09 were upregulated under LP condition but downregulated under HP condition. Some other relevant proteins including Pi-responsive proteins (B6SYB8 and A0A1D6QB24), SPX domain protein (A0A1D6HFX1) and two K^+^ transporters (A0A1D6N218 and W5UB74), and nitrate transporters (A0A1D6KAA6 and A0A1D6N629), were uniquely altered, together contributing to Pi absorption and homeostasis under different Pi conditions. A negative relationship between Na^+^ and Pi existed in plants under HP condition. Some other relevant proteins, including PAPs (B6SYB8 and A0A1D6QB24), SPX domain protein (A0A1D6HFX1) related to the regulation of PHR1 and two K^+^ transporters (A0A1D6N218 and W5UB74), and nitrate transporters (A0A1D6KAA6), were uniquely altered, together contributing to Pi absorption and homeostasis under different Pi conditions. In addition, it was shown that Pi had interactions with the micronutrients of As, Mo and B

Plants are usually subjected to Pi limitation, which hinders growth and development [3, 4, 6]. Several studies have investigated the regulated mechanism of plants response to Pi deficiency [7, 9–11], and most of them have focused on the gene changes, Few have reported on protein changes. In this study, the maize genotype QXN233, a LP-tolerant genotype as found in our previous report [52], was used to investigate the phenotypic and physiological responses to external LP and HP levels and further explore the regulated mechanism (Fig. 11). QXN233 displayed inferior and superior phenotypes under long-time LP and HP conditions, respectively (Fig. 1a, b; Additional file 1: Figs. S1, S2). The more Pi supplied, the better growth of QXN233 seedlings, as supported in various physiological indices (Fig. 1, 2; Tables 1, Additional file 1: Table S2). For instance, QXN233 accumulated MDA and proline under Pi deficiency, supported by *ZmP5CR*, *ZmP5CS*, *ZmTPS1* and *ZmSOD4* all upregulated after 12 h under LP condition, whereas MDA and proline levels were decreased under Pi sufficiency (Figs. 1c, 2c), suggesting that it recovered from LP osmotic stress. However, the protein level was low under LP condition, and no significant difference was observed in soluble sugar and SOD activity (Fig. 2d-f). This finding was probably because the plant had re-established a new balance during the long-term Pi treatment.

External Pi supplementation influenced other ion transports in plants. External Pi application has different effects on the salt tolerance of plants related to the uptake or exclusion of Na^+^ or K^+^ ion [55, 56]. In the present study, we observed high Na^+^ contents and no change in K^+^ contents. High Na^+^/K^+^ ratio emerged in shoots under LP condition, possibly indicating low salt tolerance. Importantly, in contrast to an increased Na^+^ content, a decreased Pi content was found under LP condition, while a reverse Na^+^–Pi content pattern existed under HP condition (Fig. 3a, c), showing a negative relationship between Na^+^ and Pi. Moreover, a high Na^+^ accumulation and Na^+^/K^+^ ratio appeared in roots under LP condition, although no significant difference in Pi content was observed (Fig. 3a, d). Thus, high Na^+^ in shoots might have been attributed to high Na^+^ retention in roots under LP condition. Furthermore, a high Na^+^ efflux in QXN233 root was detected under HP condition by NMT (Fig. 4a), hinting that Na^+^ efflux could be induced by external HP supplementation. Consequently, these results demonstrated a negative regulated relationship between Na^+^ and Pi, and Na^+^ exclusion could be promoted by external HP supplementation. This result can be attributed to the fact that salt tolerance may be improved by sufficient Pi application, which requires further study. If proven in the future, then external HP supplementation may be applied to improve crop.

Using RNA-Seq analysis, we previously detected several key Pi transporters that were elevated in QXN233 under Pi deficiency, such as the Pi transporter genes GRMZM2G112377, GRMZM2G070087 and GRMZM2G326707 were identified to be key genes for maize growth under LP condition [52]. Compared to our previous study, the proteomic analysis revealed that several Pi transport proteins, including Q6GUH9 (GRMZM2G326707), C4JC09 (GRMZM2G070087), and Q49B46 (GRMZM2G154090), were also upregulated under LP condition but downregulated under HP condition in the present study (Fig. 10; Table 2), implying that these proteins, especially for Q6GUH9 and C4JC09, were vital regulators for Pi absorption and transport (Fig. 11). Hence, the overexpression or RNA interference of these Pi transporter proteins was needed to verify their functions and effects on LP tolerance of maize. Moreover, Pi starvation-induced protein (B6SYB8), Pi transporter 2 (A0A1D6QB24), and SPX domain-containing membrane protein (A0A1D6HFX1) were also increased in the leaves or roots under Pi deficiency (Table 3). Previous studies revealed that SPX domain proteins regulate the expression of PHR in *Arabidopsis* and rice [31–33, 35]; however, no studies have investigated the SPX domain proteins in maize. Here, SPX domain proteins (A0A1D6HFX1) were first identified in maize response to long-term Pi deficiency, and *ZmPHR1* was also detected in roots response to LP for 12 h, suggesting that SPX protein participate in regulating the Pi signal pathway in maize (Fig. 11) and need to be further investigated next. Therefore, these proteins can be used as key candidates for gene engineering by the single or combined expression of their encoding genes in breeding tolerant maize in the future.

Finally, some cation transporters were identified and altered under LP or HP condition, especially for nitrate and potassium, indicating that Pi influenced the uptake of K and N. Other transporters, including carbohydrate, As, Mo, and B transporters, were observed, thereby implying that Pi had cross-talks with sugar metabolism and As, Mo and B transports. This result is consistent with the findings of previous studies [37, 42–48]. Therefore, Pi has a complex interplay with other nutrients and pathways, which needs to be further verified in the future.

## Conclusions

This study is the first to perform quantitative proteomic investigation to identify DEPs in maize under long-term LP and HP treatments. In-depth analysis of the 185 overlapped DEPs provided new insights into QXN233 responses to different Pi levels. Among them, Q6GUH9, Q49B46 and C4JC09 (phosphate transporters), B4F9L6 and A0A1D6KE94 (purple acid phosphatases), and A0A1D6HFX1 (SPX domain protein) associated with Pi assimilation and regulation, were increased under LP stress but decreased under HP supplementation, indicating that these DEPs play key roles in Pi homeostasis for maize coping with different Pi levels. These foundings will improve our understanding on how maize seedlings regulate DEPs to respond to different Pi environments. The key DEPs are also candidate proteins that may aid in the selection and breeding of LP-tolerant maize genotype.

## Additional file

Additional file 1: **Figure S1.** Phenotypic responses of QXN233 genotype to LP or HP condition. QXN233 grown under the different Pi-treated conditions for 10 days (a) via a vermiculite assay or for 20 days (b) via a hydroponic assay. Bar = 5 cm, Bar = 2 cm. **Figure S2.** Phenotypic responses of QXN233 genotype to LP or HP condition. QXN233 grown under the different Pi-treated conditions for 25 days (a) via a vermiculite assay. Bar = 10 cm. **Table S1.** Primers used in qRT-PCR. **Table S2.** Quantitative analyses of plant height and the width and length of the longest leaf in QXN233 after 30 days under 0 mM Pi or 3 mM Pi via vermiculite assay. Values represent means ± SEM of three replicates. Asterisks indicate a significant difference between the Pi-treated and control groups (LSD test, *P* < 0.05). **Table S3.** DEPs of QXN233 identified under low or high Pi (LP or HP) compared with the normal condition via the proteomic analysis (Ratio |0 Pi or 3 Pi/Control| > 1.2 and *P* < 0.05). The red and green markers presented the upregulated and downregulated values of DEPs, respectively. **Table S4.** Dataset.xlsx. (ZIP 4300 kb)

## Abbreviations

ABC transporter: ATP-binding cassette transporter
As: Ascorbate
B: Boron
DEPs: Differently expression proteins
FDR: False discovery rate
GO: Gene Ontology
HCD: Higher-energy collisional dissociation
HP: High Pi
KEGG: Kyoto Encyclopedia of Genes and Genomes
LC-MS/MS: Nano-liquid chromatography–mass spectrometry
LP: Low Pi
MDA: Malondialdehyde
Mo: Molybdate
MYB: Myeloblastosis viral oncogene homolog
NMT: Non-invasive micro-test technology
NPP: Nucleotide pyrophosphatase/phosphodiesterase
P: Phosphorus
P1BS: PHR1-binding sequence
P5CR: Pyrroline-5-carboxylate reductase
P5CS: Pyrroline-5-carboxylate synthetase
PAP: Purple acid phosphatase
PHO1: Phosphate 1
PHR1: Phosphate starvation response 1
PHT1: Phosphate receptor 1
Pi: Inorganic phosphate
qRT-PCR: Quantitative real-time polymerase chain reaction
SOD: Superoxide dismutase
SPX: SYG1, Pho81 and XPR1
TFs: Transcription factors
TMT: Tandem mass tag
TPS1: Trehalose-6-phosphate synthase
