# Supplementary material for: Key factors identified by proteomic analysis in maize (Zea mays L.) seedlings’ response to long-term exposure to different phosphate levels
Source: Proteome Sci. 2018 Nov 20;16:19. doi: 10.1186/s12953-018-0147-3 (PMC6247739; doi:10.1186/s12953-018-0147-3)
Supplement: Supplementary file 1 — Figure S1. Phenotypic responses of QXN233 genotype to LP or HP condition. QXN233 grown under the different Pi-treated conditions for 10 days (a) via a vermiculite assay or for 20 days (b) via a hydroponic assay. Bar = 5 cm, Bar = 2 cm. Figure S2. Phenotypic responses of QXN233 genotype to LP or HP condition. QXN233 grown under the different Pi-treated conditions for 25 days (a) via a vermiculite assay. Bar = 10 cm. Table S1. Primers used in qRT-PCR. Table S2. Quantitative analyses of plant height and the width and length of the longest leaf in QXN233 after 30 days under 0 mM Pi or 3 mM Pi via vermiculite assay. Values represent means ± SEM of three replicates. Asterisks indicate a significant difference between the Pi-treated and control groups (LSD test, P < 0.05). Table S3. DEPs of QXN233 identified under low or high Pi (LP or HP) compared with the normal condition via the proteomic analysis (Ratio |0 Pi or 3 Pi/Control| > 1.2 and P < 0.05). The red and green markers presented the upregulated and downregulated values of DEPs, respectively. Table S4. Dataset.xlsx. (ZIP 4300 kb) [file 12953_2018_147_MOESM1_ESM.zip › Table S1.docx]

**Table S1.** Primers used in qRT-PCR.

| Gene name | Genbank accession number | Forward/reverse primer | Sequence (5’-3’) | Product  (bp) |
| --- | --- | --- | --- | --- |
| *18S rRNA* | AF168884 | Forward | CCATCCCTCCGTAGTTAGCTTCT | 151 bp |
|  |  | Reverse | CCTGTCGGCCAAGGCTATATAC |  |
| *ZmPHR1* | JF831533 | Forward | CACCCTTTATTTCTCAGTCATCCAA | 205 bp |
|  |  | Reverse | TCATTTTGTGTAGCACTCTCATCAG |  |
| *ZmPhytase2* | AJ223471 | Forward | CGGGCAACCTGGCGTGG | 134 bp |
|  |  | Reverse | CGCCTTGGTGACAGCCGC |  |
| *ZmPHT1;3* | GRMZM2G112377 | Forward | CGTCCTCATCCCTCGCTGC | 93 bp |
|  |  | Reverse | CCCCGCCTCAACCTGTCTC |  |
| *ZmPHT1;4* | GRMZM2G170208 | Forward | TGGCGTGTTTGCCTTCGTT | 136 bp |
|  |  | Reverse | CTCCTCCAGTGCCTTCCCCT |  |
| *ZmPHT1;8* | GRMZM2G045473 | Forward | CATTGTCGGGTCATTTGGGTTC | 207 bp |
|  |  | Reverse | GCCTCCTCGTCGTTCTCGC |  |
| *ZmPHT1; 9*  /*ZmPHT2* | GRMZM2G154090 | Forward | ACGACCACTTCAACTCCACCG | 157 bp |
|  |  | Reverse | GCCTGCTTGGCGTTCTTGG |  |
| *ZmP5CS* | DQ864376 | Forward | GCGAGGAAGTGGGCAAGTGGT | 250 bp |
|  |  | Reverse | TTGGGGAGGTGGGGTGGC |  |
| *ZmP5CR* | DQ026301 | Forward | CCAGCCTGTGCCAACCGC | 147 bp |
|  |  | Reverse | GTGCGGATGGCGGAGGC |  |
| *ZmTPS1* | AF529266 | Forward | GGTTGCAGCGTTTCCTATTG | 177 bp |
|  |  | Reverse | AATCAAGAGATCGGTCCAGATG |  |
| *ZmSOD4* | XM_008650839 | Forward | TAAGCACCTGTGGCAACCGAT | 115 bp |
|  |  | Reverse | ACGAAACGGTCGGAATGCC |  |
| *ZmPHT1* | GRMZM2G326707 | Forward | TCCTCGCCGCCAGCAACAT | 119 bp |
|  |  | Reverse | GCCGACGGGCTCCTCCTCT |  |
| *ZmPHT9* | GRMZM2G070087 | Forward | TACGCCAACAAGAAGACCCG | 141 bp |
|  |  | Reverse | CACGAAGTAGGGCGGGG |  |
| *ZmPAP10* | GRMZM2G093101 | Forward | CACGCCATCCTGGACATCAAGA | 223 bp |
|  |  | Reverse | CTTCGTGTATTCAGGGCGATGCT |  |
| *ZmNPP* | GRMZM2G315848 | Forward | GAACGGCTACTTGTCTCAGTGGGA | 248 bp |
|  |  | Reverse | GAGCGACGCAGAACCTGAACAT |  |
| *ZmSPX* | GRMZM2G166976 | Forward | GCTCCTGGCTTTGGTGCCGTAT | 181 bp |
|  |  | Reverse | GGCGTGCGGCTCGTCAACT |  |
