# Supplementary material for: Key factors identified by proteomic analysis in maize (Zea mays L.) seedlings’ response to long-term exposure to different phosphate levels
Source: Proteome Sci. 2018 Nov 20;16:19. doi: 10.1186/s12953-018-0147-3 (PMC6247739; doi:10.1186/s12953-018-0147-3)
Supplement: Supplementary file 1 — Figure S1. Phenotypic responses of QXN233 genotype to LP or HP condition. QXN233 grown under the different Pi-treated conditions for 10 days (a) via a vermiculite assay or for 20 days (b) via a hydroponic assay. Bar = 5 cm, Bar = 2 cm. Figure S2. Phenotypic responses of QXN233 genotype to LP or HP condition. QXN233 grown under the different Pi-treated conditions for 25 days (a) via a vermiculite assay. Bar = 10 cm. Table S1. Primers used in qRT-PCR. Table S2. Quantitative analyses of plant height and the width and length of the longest leaf in QXN233 after 30 days under 0 mM Pi or 3 mM Pi via vermiculite assay. Values represent means ± SEM of three replicates. Asterisks indicate a significant difference between the Pi-treated and control groups (LSD test, P < 0.05). Table S3. DEPs of QXN233 identified under low or high Pi (LP or HP) compared with the normal condition via the proteomic analysis (Ratio |0 Pi or 3 Pi/Control| > 1.2 and P < 0.05). The red and green markers presented the upregulated and downregulated values of DEPs, respectively. Table S4. Dataset.xlsx. (ZIP 4300 kb) [file 12953_2018_147_MOESM1_ESM.zip › Table S2.docx]

**Table S2.** Quantitative analyses of plant height, leaf-width and leaf-length of the longest leaf in QXN233 after 30 d under 0 mM Pi or 3 mM Pi by the vermiculite assay. Values represent means ± SD of three replicates. Asterisks indicate a significant difference between the Pi-treated and control group (*t*-test, P < 0.05).

| QXN233 | plant height (cm) | leaf-width of the longest leaf (cm) | leaf-length of the longest leaf (cm) |
| --- | --- | --- | --- |
| 0 mM Pi | 34.38^*^ ± 2.8 | 1.93^*^ ± 0.3 | 34.75^*^ ± 4.8 |
| Control | 59.75 ± 0.3 | 2.65 ± 0.3 | 50.75 ± 0.3 |
| 3 mM Pi | 60.00 ± 8.4 | 3.30^*^ ± 0.1 | 59.00^*^ ± 2.1 |
