# Supplementary material for: Key factors identified by proteomic analysis in maize (Zea mays L.) seedlings’ response to long-term exposure to different phosphate levels
Source: Proteome Sci. 2018 Nov 20;16:19. doi: 10.1186/s12953-018-0147-3 (PMC6247739; doi:10.1186/s12953-018-0147-3)
Supplement: Supplementary file 1 — Figure S1. Phenotypic responses of QXN233 genotype to LP or HP condition. QXN233 grown under the different Pi-treated conditions for 10 days (a) via a vermiculite assay or for 20 days (b) via a hydroponic assay. Bar = 5 cm, Bar = 2 cm. Figure S2. Phenotypic responses of QXN233 genotype to LP or HP condition. QXN233 grown under the different Pi-treated conditions for 25 days (a) via a vermiculite assay. Bar = 10 cm. Table S1. Primers used in qRT-PCR. Table S2. Quantitative analyses of plant height and the width and length of the longest leaf in QXN233 after 30 days under 0 mM Pi or 3 mM Pi via vermiculite assay. Values represent means ± SEM of three replicates. Asterisks indicate a significant difference between the Pi-treated and control groups (LSD test, P < 0.05). Table S3. DEPs of QXN233 identified under low or high Pi (LP or HP) compared with the normal condition via the proteomic analysis (Ratio |0 Pi or 3 Pi/Control| > 1.2 and P < 0.05). The red and green markers presented the upregulated and downregulated values of DEPs, respectively. Table S4. Dataset.xlsx. (ZIP 4300 kb) [file 12953_2018_147_MOESM1_ESM.zip › Table S3.docx]

**Table S3** The differentially expressed proteins (DEPs) of QXN233 identified under low Pi (LP) or high Pi (HP) compared to the normal condition by the proteomic analysis (Ratio |0 Pi or 3 Pi/Control| > 1.2 and P value < 0.05). The red and green markers presented the upregulated and downregulated value of DEPs, respectively.

| Accession | | Description | L-(0Pi/Control) | L-(3Pi/Control) | R-(0Pi/Control) | R-(3Pi/Control) |
| --- | --- | --- | --- | --- | --- | --- |
| **Metabolism** | |  |  |  |  |  |
| A0A1D6I1V3 | | Phosphoenolpyruvate carboxylase3 | 1.73 |  | 1.33 | 0.80 |
| A0A1D6PRE0 | | Putative sucrose-phosphate synthase family protein | 1.62 |  | 2.14 | 0.82 |
| A0A1D6H4D6 | | Citrate synthase | 1.50 |  |  |  |
| B6U167 | | Starch synthase, chloroplastic/amyloplastic | 1.50 |  |  |  |
| P08440 | | Fructose-bisphosphate aldolase, cytoplasmic isozyme | 1.41 |  |  |  |
| A0A1D6GBA2 | | Phosphoenolpyruvate carboxylase 3 | 1.39 |  |  |  |
| P31927 | | Sucrose-phosphate synthase | 1.37 |  |  |  |
| B7ZZ56 | | Glycosyltransferase | 1.36 |  |  |  |
| Q2XX18 | | Non-specific lipid-transfer protein (Fragment) | 1.35 | 1.33 |  |  |
| B6UGU8 | | Indole-3-glycerol phosphate synthase | 1.27 |  |  |  |
| A0A1D6GWZ8 | | Sucrose synthase |  |  | 1.33 |  |
| **Photosynthesis** | |  |  |  |  |  |
| B4FYS2 | | Cytochrome c-2 | 1.65 |  |  | 1.85 |
| C0PDM0 | | Pyrophosphate-energized vacuolar proton pump 1 | 1.59 |  |  |  |
| B4FAD9 | | UDP-glucose pyrophosphorylase2 | 1.57 |  |  |  |
| B6TGS7 | | Cytochrome c | 1.52 | 1.29 |  | 1.32 |
| B6SKR4 | | Cytochrome c | 1.48 | 1.29 |  | 1.78 |
| B6SUE5 | | Serine/threonine kinase-like protein | 1.44 | 1.28 |  |  |
| O65101 | | Photosystem I reaction center subunit VI, chloroplastic | 1.38 |  |  |  |
| C0P9F5 | | Chlorophyll a-b binding protein, chloroplastic | 1.38 |  |  |  |
| B4FQ80 | | Chlorophyll a-b binding protein, chloroplastic | 1.38 |  |  |  |
| B6STP9 | | Photosystem I reaction center subunit XI | 1.32 |  |  |  |
| Q9SLP6 | | Ferredoxin | 1.28 |  |  |  |
| B4FUA1 | | Chlorophyll a-b binding protein, chloroplastic | 1.27 |  |  |  |
| B4FRC8 | | FAD/NAD(P)-binding oxidoreductase | 1.27 |  |  |  |
| B4FQI3 | | NADH dehydrogenase 1 alpha subcomplex subunit 13-A |  |  | 1.35 |  |
| A0A1D6E9A6 | | NAD(P)-binding Rossmann-fold superfamily protein |  |  |  | 1.22 |
| A0A1D6KWI5 | | Chlorophyll a-b binding protein, chloroplastic | 0.70 | 0.69 |  |  |
| B4F9W3 | | Chlorophyll a-b binding protein, chloroplastic | 0.71 | 0.70 |  |  |
| B6ST66 | | Cytochrome P450 CYP92C5 | 0.74 | 0.80 |  |  |
| A0A1D6IX37 | | Cytochrome b5 isoform B | 0.76 | 0.81 |  |  |
| **ATP metabolism** | | |  |  |  |  |
| B4FPE4 | | V-type proton ATPase subunit G | 1.44 | 1.21 | 1.58 | 1.48 |
| K7TX67 | | Plasma membrane ATPase | 1.43 |  | 1.56 | 0.78 |
| A0A1D6HUU8 | | Plasma membrane ATPase | 1.36 |  |  |  |
| B8A326 | | Plasma membrane ATPase | 1.31 |  |  |  |
| A0A1D6MYY5 | | V-type proton ATPase proteolipid subunit | 1.31 |  |  |  |
| A0A059Q8F8 | | ATP synthase subunit b, chloroplastic | 1.29 |  |  |  |
| P69449 | | ATP synthase subunit c, chloroplastic | 1.28 |  |  |  |
| **Transcriptional regulators** | | |  |  |  |  |
| A0A1D6JNC8 | | Zinc finger (C3HC4-type RING finger) family protein | 2.65 |  | 1.58 |  |
| K7TP80 | | Zinc finger (C3HC4-type RING finger) family protein | 1.31 |  |  |  |
| K7UGV0 | | Zn-finger, RanBP-type, containing protein | 1.30 |  |  |  |
| A0A1D6H6V1 | | Calcium-binding EF hand family protein | 0.70 |  |  |  |
| K7V2L1 | | Putative RING zinc finger domain superfamily protein | 0.75 | 0.68 |  |  |
| A0A1D6GWN0 | | General transcription factor 2-related zinc finger protein | 0.79 | 1.24 |  |  |
| K7VWP5 | | Basic leucine zipper 24 |  |  | 3.36 |  |
| **Translation** | |  |  |  |  |  |
| B6T329 | | Endonuclease 2 | 5.81 | 0.77 |  | 0.76 |
| B4FRP4 | | Endonuclease 2 | 2.16 |  |  |  |
| C0HHC4 | | Nucleoside diphosphate kinase | 1.55 |  | 1.37 |  |
| A0A1D6FFG6 | | Eukaryotic translation initiation factor 3 subunit A | 1.45 |  |  |  |
| A0A1D6LJL2 | | UMP/CMP kinase1 | 1.42 |  | 1.80 |  |
| B4FIA6 | | Histone H2A | 1.33 | 1.59 |  |  |
| B4FJK0 | | Histone H2A | 1.31 | 1.21 |  |  |
| Q8L8G5 | | Nucleosome assembly protein 1 | 1.31 |  | 1.24 |  |
| B6UFX4 | | Histone H2B |  |  | 1.33 | 0.42 |
| A0A1D6EXC9 | | 60S ribosomal protein L44 | 0.63 | 1.23 |  |  |
| B4FW18 | | 40S ribosomal protein S28 | 0.66 | 0.69 |  |  |
| B6SLI1 | | 40S ribosomal protein S30 | 0.68 |  |  | 0.63 |
| P25460 | | 40S ribosomal protein S11 | 0.68 |  | 0.65 | 1.22 |
| B6T0Z9 | | 60S ribosomal protein L27a-3 | 0.69 |  |  | 0.60 |
| B4FP25 | | 40S ribosomal protein S19 | 0.69 |  | 0.72 |  |
| B4FL64 | | Ribosomal protein L19 | 0.70 |  |  | 1.27 |
| B6SYS9 | | 60S ribosomal protein L30 | 0.70 |  | 0.60 |  |
| B6U0S1 | | Elongation factor 2 | 0.70 |  | 0.74 |  |
| A0A1D6PW12 | | 60S ribosomal protein L34-3 | 0.71 |  | 0.77 | 1.33 |
| K7TT73 | | 40S ribosomal protein S3-1 | 0.73 |  | 0.81 |  |
| B6SIX3 | | 40S ribosomal protein S24 | 0.73 | 1.25 | 0.68 |  |
| B4FHM5 | | 60S ribosomal protein L37a | 0.73 |  | 0.68 | 1.31 |
| B6SSB6 | | Eukaryotic translation initiation factor 3 subunit I | 0.74 |  | 0.81 |  |
| B4FUA9 | | 60 ribosomal protein L14 | 0.74 |  | 0.69 |  |
| B6SGI4 | | Ribosomal protein L37 | 0.74 |  | 0.59 | 1.28 |
| B4FCK9 | | 60S ribosomal protein L22-2 | 0.74 |  | 0.68 | 1.20 |
| **Cell growth** | |  |  |  |  |  |
| A0A1D6ML43 | | Cell number regulator 8 | 1.88 |  |  | 0.83 |
| K7W5K6 | | Pectinesterase | 0.70 | 0.78 |  |  |
| A0A077D360 | | Cellulose synthase | 0.70 | 0.62 |  |  |
| B6TH29 | | Cell division protein ftsZ | 0.75 |  |  |  |
| **Phytohormone regulation** | | |  |  |  |  |
| A0A1D6EB22 | Abscisic acid stress ripening3 | | 4.69 | 0.83 | 4.04 | 0.42 |
| D1MN58 | ABA-, stress-and fruit-ripening inducible-like protein | | 1.36 |  |  |  |
| A0A0F7GPD7 | Brassinosteroid receptor | | 1.31 |  |  |  |
| B6TKK2 | Gibberellin receptor GID1L2 | | 1.31 |  | 1.25 |  |
| A0A1D6HB17 | Gibberellin receptor GID1L2 | | 0.78 |  |  |  |
| B6SV38 | BR INSENSITIVE 1-associated receptor kinase 1 | |  |  | 1.37 |  |
| **Oxidation-reduction process** | | |  |  |  |  |
| B4FA32 | Peroxidase | | 2.48 | 1.47 |  |  |
| B8A1T1 | Peroxidase | |  |  | 1.33 |  |
| K7V8K5 | Peroxidase | | 1.92 |  |  |  |
| C4J6E4 | Peroxidase | | 1.83 |  |  |  |
| A0A1D6GUG0 | Peroxidase | |  |  | 1.40 |  |
| A0A1D6LE55 | Peroxidase | | 1.46 |  | 1.93 |  |
| B6THG0 | Peroxidase | | 1.36 |  |  |  |
| B6TMI9 | Peroxidase | | 1.28 |  |  |  |
| B6TIS2 | Superoxide dismutase [Cu-Zn] | | 1.53 |  | 1.43 |  |
| P11428 | Superoxide dismutase [Cu-Zn] 2 | | 1.50 |  |  |  |
| B4F9H6 | Superoxide dismutase | | 1.28 |  | 1.38 |  |
| Q9FQA3 | Glutathione transferase GST 23 | | 1.87 |  | 1.64 |  |
| O24595 | Glutathione transferase | | 1.40 |  |  |  |
| B6SS87 | Glutathione S-transferase GSTU6 | | 1.39 |  |  |  |
| Q9FQC2 | Glutathione S-transferase GST 17 | | 1.33 |  | 1.50 |  |
| B6SHW5 | Thioredoxin | | 1.46 |  |  |  |
| B6SNA1 | Putative thioredoxin superfamily protein | | 1.31 |  | 1.74 |  |
| A0A1D6HLS2 | Thioredoxin-like 3-1 chloroplastic | | 1.27 |  |  |  |
| B6T2J0 | Thioredoxin | |  |  | 1.90 | 0.69 |
| C0P4M0 | Monodehydroascorbate reductase 1 peroxisomal | | 1.42 |  |  |  |
| K7UZN5 | Putative oxidoreductase, aldo/keto reductase family protein | | 1.30 |  |  |  |
| C4J4E4 | Monodehydroascorbate reductase homolog1 | | 1.30 |  | 1.36 | 0.82 |
| C4J9K0 | APX3-Peroxisomal Ascorbate Peroxidase | |  |  | 1.41 |  |
| A0A1D6EAC4 | L-ascorbate peroxidase S chloroplastic/mitochondrial | |  |  | 1.38 |  |
| **Stress response** | | |  |  |  |  |
| A0A1D6LGF5 | Metal tolerance protein A2 | | 3.50 |  | 2.45 |  |
| A0A1D6EDE3 | Metal tolerance protein C4 | | 1.38 |  |  |  |
| A0A1D6FV33 | Metal tolerance protein A2 | | 1.36 | 0.79 | 1.97 |  |
| B6TV55 | Stem 28 kDa glycoprotein | | 2.44 |  |  |  |
| B6T003 | Stem 28 kDa glycoprotein | | 1.66 |  |  |  |
| B4FV56 | Osmotin-like protein OSM34 | | 1.63 | 1.29 | 1.33 |  |
| C0P6C7 | HSP40/DnaJ peptide-binding protein | | 1.53 |  |  |  |
| B6TMF3 | Wound/stress protein | | 1.97 |  | 1.69 | 0.78 |
| A0A1D6FUF5 | Stress protein | | 1.33 |  | 1.44 |  |
| B6TFB6 | Stress responsive protein | |  |  | 1.22 |  |
| Q9FYU0 | Calcium/calmodulin dependent protein kinase MCK2 | |  |  | 1.23 | 0.81 |
| A0A1D6IGW8 | Calcium-dependent protein kinase 7 | |  |  | 1.21 |  |
| C0P3S3 | Calreticulin-3 | | 1.27 |  |  |  |
| K7VJF3 | Heat shock 70 kDa protein 5 | | 1.40 |  |  |  |
| B6U3T7 | Aquaporin TIP2.1 | |  |  |  | 0.65 |
| Q9ATN0 | Aquaporin PIP1-6 | |  |  | 0.58 |  |
| Q9AQU5 | Aquaporin PIP1-3/PIP1-4 | | 1.34 |  |  |  |
| B6TBQ1 | Hypersensitive-induced response protein | | 1.35 |  |  |  |
| B6T9C5 | Senescence-associated protein | | 1.34 |  |  |  |
| A0A1D6IAX6 | High mobility group B protein 2 | | 0.78 |  | 0.61 |  |
