# Supplementary figures and images for: Key factors identified by proteomic analysis in maize (Zea mays L.) seedlings’ response to long-term exposure to different phosphate levels
Source: Proteome Sci. 2018 Nov 20;16:19. doi: 10.1186/s12953-018-0147-3 (PMC6247739; doi:10.1186/s12953-018-0147-3)

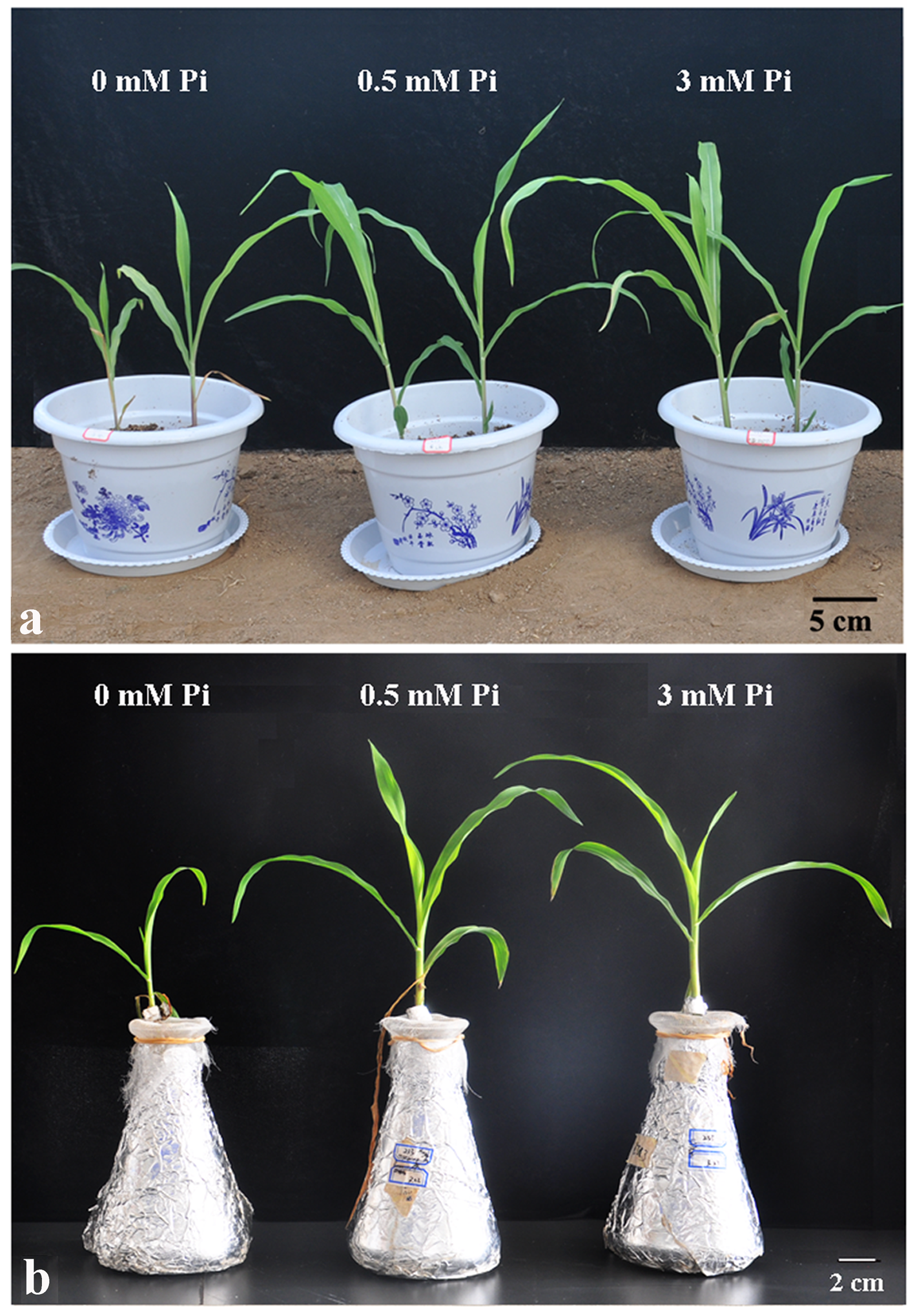

Supplement: Supplementary file 1 — Figure S1. Phenotypic responses of QXN233 genotype to LP or HP condition. QXN233 grown under the different Pi-treated conditions for 10 days (a) via a vermiculite assay or for 20 days (b) via a hydroponic assay. Bar = 5 cm, Bar = 2 cm. Figure S2. Phenotypic responses of QXN233 genotype to LP or HP condition. QXN233 grown under the different Pi-treated conditions for 25 days (a) via a vermiculite assay. Bar = 10 cm. Table S1. Primers used in qRT-PCR. Table S2. Quantitative analyses of plant height and the width and length of the longest leaf in QXN233 after 30 days under 0 mM Pi or 3 mM Pi via vermiculite assay. Values represent means ± SEM of three replicates. Asterisks indicate a significant difference between the Pi-treated and control groups (LSD test, P < 0.05). Table S3. DEPs of QXN233 identified under low or high Pi (LP or HP) compared with the normal condition via the proteomic analysis (Ratio |0 Pi or 3 Pi/Control| > 1.2 and P < 0.05). The red and green markers presented the upregulated and downregulated values of DEPs, respectively. Table S4. Dataset.xlsx. (ZIP 4300 kb) [file 12953_2018_147_MOESM1_ESM.zip › Fig. S1.tif]

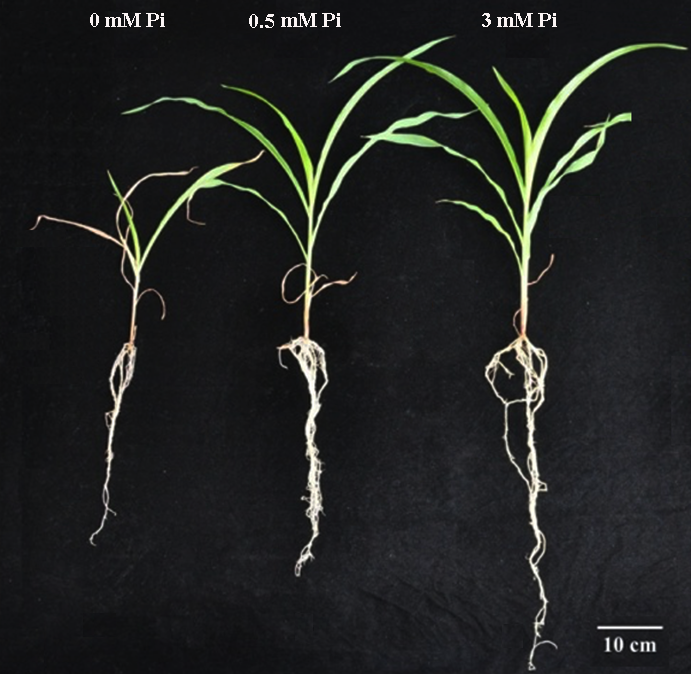

Supplement: Supplementary file 1 — Figure S1. Phenotypic responses of QXN233 genotype to LP or HP condition. QXN233 grown under the different Pi-treated conditions for 10 days (a) via a vermiculite assay or for 20 days (b) via a hydroponic assay. Bar = 5 cm, Bar = 2 cm. Figure S2. Phenotypic responses of QXN233 genotype to LP or HP condition. QXN233 grown under the different Pi-treated conditions for 25 days (a) via a vermiculite assay. Bar = 10 cm. Table S1. Primers used in qRT-PCR. Table S2. Quantitative analyses of plant height and the width and length of the longest leaf in QXN233 after 30 days under 0 mM Pi or 3 mM Pi via vermiculite assay. Values represent means ± SEM of three replicates. Asterisks indicate a significant difference between the Pi-treated and control groups (LSD test, P < 0.05). Table S3. DEPs of QXN233 identified under low or high Pi (LP or HP) compared with the normal condition via the proteomic analysis (Ratio |0 Pi or 3 Pi/Control| > 1.2 and P < 0.05). The red and green markers presented the upregulated and downregulated values of DEPs, respectively. Table S4. Dataset.xlsx. (ZIP 4300 kb) [file 12953_2018_147_MOESM1_ESM.zip › Fig. S2.tif]
